# Investigation of Thermomorphogenesis-Related Genes for a Multi-Silique Trait in *Brassica napus* by Comparative Transcriptome Analysis

**DOI:** 10.3389/fgene.2021.678804

**Published:** 2021-07-23

**Authors:** Liang Chai, Jinfang Zhang, Haojie Li, Cheng Cui, Jun Jiang, Benchuan Zheng, Lintao Wu, Liangcai Jiang

**Affiliations:** ^1^Crop Research Institute, Sichuan Academy of Agricultural Sciences, Chengdu, China; ^2^School of Biological Sciences, Guizhou Education University, Guiyang, China

**Keywords:** *Brassica napus*, differentially expressed gene, environmental effect, multi-silique, RNA-seq

## Abstract

In higher plants, the structure of a flower is precisely controlled by a series of genes. An aberrance flower results in abnormal fruit morphology. Previously, we reported multi-silique rapeseed (*Brassica napus*) line zws-ms. We identified two associated regions and investigated differentially expressed genes (DEGs); thus, some candidate genes underlying the multi-silique phenotype in warm area Xindu were selected. However, this phenotype was switched off by lower temperature, and the responsive genes, known as thermomorphogenesis-related genes, remained elusive. So, based on that, in this study, we further investigated the transcriptome data from buds of zws-ms and its near-isogenic line zws-217 grown in colder area Ma’erkang, where both lines showed normal siliques only, and the DEGs between them analyzed. We compared the 129 DEGs from Xindu to the 117 ones from Ma’erkang and found that 33 of them represented the same or similar expression trends, whereas the other 96 DEGs showed different expression trends, which were defined as environment-specific. Furthermore, we combined this with the gene annotations and ortholog information and then selected BnaA09g45320D (chaperonin gene *CPN10*-homologous) and BnaC08g41780D [Seryl-tRNA synthetase gene *OVULE ABORTION 7* (*OVA7*)-homologous] the possible thermomorphogenesis-related genes, which probably switched off the multi-silique under lower temperature. This study paves a way to a new perspective into flower/fruit development in *Brassica* plants.

## Introduction

Flower development, as well as the subsequent fruit formation, is vital to the crop life cycle. Siliques are important to rapeseed (*Brassica napus*, AACC = 38), the leading source of plant oil worldwide, which offers more than 13% of the global vegetable oil (Geng et al., 2016). In rapeseed, siliques supply nutrients from photosynthesis, transport carbohydrates from the vegetative organs to the seeds, and ensure their development (Shen et al., 2019). In addition, silique number is a crucial component determining seed yield per plant (Yang et al., 2012; Wang et al., 2016; Shen et al., 2019).

We previously reported a multi-silique phenotype in rapeseed line zws-ms (Chai et al., 2019, 2020a,b), which presents three pistils instead of one typical pistil in flower, and then develops three siliques on a carpopodium. This trait is different from the multilocular phenotype in *Brassica* plants, such as tetra-locular *Brassica rapa* (Yadava et al., 2014; Lee et al., 2018), multilocular *Brassica juncea* (Xiao et al., 2013; Xu et al., 2014), etc., which increases the number of locules in a silique. However, similar phenomena described as “multi-pistil” have been reported in wheat (*Triticum aestivum*) (Yang et al., 2011, 2017; Duan et al., 2015; Wei, 2017; Guo et al., 2019; Zhu et al., 2019; Yu et al., 2020), rice (*Oryza sativa*) (Zheng et al., 2019), sweet cherry (*Prunus avium*) (Liu et al., 2019; Wang et al., 2019), and alfalfa (*Medicago sativa*) (Nair et al., 2008). In wheat, increasing the number of grains per spike is considered to be vital for maximizing its yield potential (Wei, 2017; Zhu et al., 2019; Yu et al., 2020). The multi-pistil traits in wheat were found to be controlled by a recessive gene, two recessive non-complementary genes, or a single dominant gene; F_2_ populations, BC_6_F_2_ populations, or near-isogenic lines (NILs) were constructed to map the underlying locus, and they found to be located on 2DL, 5DS, 6BS, and 6B (Zhu et al., 2019). However, there has been no gene cloned so far.

The environment can influence various aspects of plants. Temperature, a key environmental factor, affects the growth, development, and geographical distribution of plants, as well as the quality and productivity of crops (Ding et al., 2020). Therein, “thermomorphogenesis” is defined as the effect of temperature on the morphogenesis of plants (Casal and Balasubramanian, 2019). Take soybean (*Glycine max*) for example; a 4°C rise in the temperature could increase its stem height more than 3-fold (Sionit et al., 1987); additionally, the temperature raised from 10 to 15°C could increase the leaf number in wheat (*T. aestivum*) (Friend, 1965), whereas, in model plant *Arabidopsis thaliana*, higher temperatures reduced both silique number per plant and seed number per silique (Ibañez et al., 2017).

Similarly, as described in earlier studies (Chai et al., 2019, 2020a), the multi-silique trait in zws-ms is significantly affected by the environment. Precisely, zws-ms showed stable multi-silique trait in Xindu (with an annual average temperature of 16.2°C) for successive years; however, when grown in a colder area such as Ma’erkang (with an annual average temperature of 8.6°C), zws-ms switched off the formation of multi-silique, and all siliques appeared normal. In previous studies, we identified two associated regions on chromosome A09 and C08 and screened out some potential candidate genes by the combination of bulked-segregant analysis and whole-genome re-sequencing; we also analyzed genes differentially expressed between NILs zws-ms (multi-silique) and zws-217 (normal silique) from Xindu and selected potential underlying genes based on annotations. However, the transcriptome from Ma’erkang, where the multi-silique morphology is switched off, as well as the comparison of differentially expressed genes (DEGs) between Xindu and Ma’erkang, remains unclear. In other words, genes involved in the thermomorphogenesis of this multi-silique trait require further investigation.

Thus, in this study, we identified DEGs based on transcriptome sequencing (RNA-seq) from Ma’erkang and then compared them with those from Xindu. The variations in DEGs, combined with their annotations and information of orthologs in *Arabidopsis*, were then analyzed, and then some potential underlying genes related to thermomorphogenesis were identified.

## Materials and Methods

### Plant Materials, Growth Conditions, and Sample Collection

The rapeseed line zws-ms and its NIL, zws-217, were kept in the Crop Research Institute, Sichuan Academy of Agricultural Sciences, China. The multi-silique plant was originally discovered in progenies of *B. napus* × *B. rapa* and was successively self-pollinated for six successive generations until the homozygous multi-silique zws-ms line was obtained, whereas the single-silique offspring were continuously backcrossed with zws-ms (current parent) for six generations, followed by six continuous generations of self-pollination until zws-217 with the single-silique was obtained. The two lines were simultaneously grown in September in Xindu District of Chengdu in the Sichuan Basin under normal environmental conditions (with an annual average temperature of 16.2°C). Moreover, both lines were also grown concurrently in early June in Ma’erkang, a mountainous area of western Sichuan, with an annual average temperature of 8.6°C.

In each location, buds were sampled at their budding stage. In Xindu, three randomly selected individual plants of the zws-ms were assigned as T01, T02, and T03, and three plants in the zws-217 line were assigned as T04, T05, and T06, whereas in colder area Ma’erkang, plants in zws-ms were defined as T01′, T02′, and T03′, and plants in zws-217 were defined as T04′, T05′, and T06′. Eight to 10 buds were sampled from each plant and then quick-frozen and stored in liquid nitrogen.

### RNA Isolation and the Library Preparation

The total RNA was isolated as Chai et al. (2019) described. The OD260/OD230 value and concentration were determined on NanoDrop 2000 (Thermo Fisher Scientific, Waltham, MA, United States) for the quality control of RNA. The sequencing libraries were generated by RNA Library Prep Kit for Illumina (New England Biolabs, Ipswich, MA, United States), according to its instructions.

### Transcriptome Sequencing

The transcriptome sequencing (RNA-seq) was performed on the Illumina HiSeq X-ten platform. In-house Perl scripts were used to remove adapter sequences and read containing poly-*N*, or low-quality reads, to process the initially generated raw reads into clean reads. Clean reads were then aligned to the *B. napus* “Darmor-*bzh*” reference genome^1^ by using Tophat2 tools (Kim et al., 2013) to screen out the reads with a perfect match or one mismatch for the next investigation.

### Differentially Expressed Gene Analysis

DEGs were detected by the DESeq R package (Love et al., 2014). The *P*-value was adjusted by controlling the false discovery rate (FDR) (Benjamini and Hochberg, 1995), and genes with an adjusted fold change (FC) > 4 (log_2_FC > 2) and an FDR < 0.001 were then identified as DEGs.

### Annotation of Genes

Gene Ontology (GO) database^2^ and GOseq R packages (Young et al., 2010) were used to provide gene annotations and calculate GO enrichment of the DEGs. The Kyoto Encyclopedia of Genes and Genomes (KEGG) database^3^ and KOBAS software (Mao et al., 2005) were used to explore the high-level functions and utilities of the biological system (Kanehisa et al., 2007) and test the statistical enrichment of the DEGs in the various KEGG pathways. The TAIR database^4^ was utilized to provide ortholog information from the model plant *Arabidopsis*. Sequences of genes from rapeseed were blasted on the website, and orthologs in *Arabidopsis* were then screened out. Orthologs in *Arabidopsis* were sufficiently annotated and able to give abundant information, as they have been well studied in the model plant and reported.

## Results

### Multi-Silique Trait Under Normal Conditions and Its Absence in Colder Areas

**FIGURE 1:**
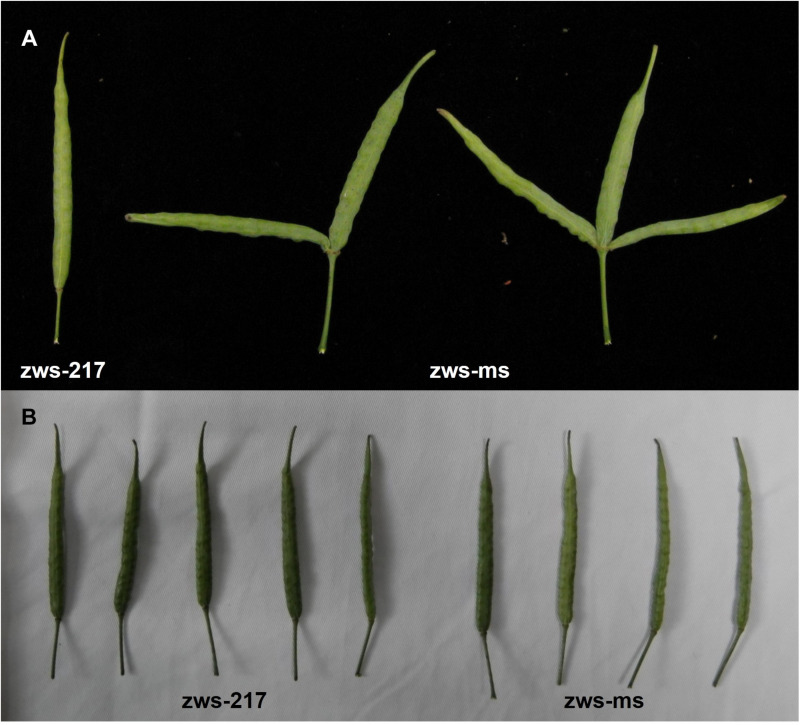
Multi-silique phenotype in zws-ms. **(A)** Compared with normal silique in its near-isogenic line (NIL) zws-217, zws-ms had three individual siliques sharing one carpopodium in Xindu. Sometimes, one of silique degrade. **(B)** Both zws-ms and zws-217 showed normal siliques in colder Ma’erkang.

Compared with its NIL zws-217 with normal siliques, the zws-ms line displayed stable multi-pistil phenotype in successive years in Xindu, where a zws-ms plant showed approximately 32–53% of flowers with the multi-pistil trait, and that further developed into a multi-silique trait, precisely, two or three siliques on a carpopodium (Figure 1A), like we previously described (Chai et al., 2019). When grown in Ma’erkang, a colder place in a mountain area of western Sichuan Province, zws-ms switched off this multi-silique phenotype; in other words, both lines produced normal siliques there (Figure 1B).

### Transcriptome Sequencing (RNA-Seq) From Two Environments

As described previously (Chai et al., 2019, 2020a), the two lines were grown at Xindu and Ma’erkang. At each place, flower buds from three individual plants from each line were selected randomly for RNA isolation. Then, their total RNA was extracted, and sequencing libraries were generated, followed by being sequenced on a HiSeq X-Ten platform; the validation for the RNA-seq was also confirmed by real-time quantitative polymerase chain reaction (Chai et al., 2019, 2020a).

### Comparison of Differentially Expressed Genes Between Two Environments

Genes in an environment with expression level fold change > 4 (log_2_FC > 2) between zws-ms and zws-217 and FDR < 0.001 were identified as DEGs. In our earlier report (Chai et al., 2019), 129 DEGs were found between zws-ms and zws-217 in Xindu, among which 67 genes were upregulated, whereas 62 were downregulated. In this study, both lines were grown in colder area Ma’erkang, where they did not show any phenotypic differences to each other and were further subjected to RNA-seq, and 117 genes were found expressed differentially in stamen and pistils between zws-ms and zws-217 (Table 1), including 63 upregulated and 54 downregulated in zws-ms (Supplementary Table 1). Samples from the two environments generated different DEGs between zws-ms and zws-217, but in either environment, chromosome A09 and C08, where two associated regions (Chai et al., 2019) underlying this trait were identified, provided most DEGs: in Xindu, 16 (12.4%) and 30 (23.26%) genes were located on chromosome A09 and C08, respectively, whereas 7 (5.98%) and 23 (19.66%) genes were found on chromosome A09 and C08, respectively, of samples from Ma’erkang.

**TABLE 1:** Number of differentially expressed genes between zws-ms and zws-217 in two environments.

	Number of DEGs	Upregulated genes	Downregulated genes
Xindu	129	67	62
Ma’erkang	117	63	54

Further analysis found that among these DEGs, there were some expressed only in zws-ms or zws-217, which were assigned “line-specific expressed genes” in this study. Herein, we discovered 21 genes that were line-specific expressed in zws-ms and 25 in zws-217 from Xindu (Supplementary Table 2), whereas from Ma’erkang, 18 and one line-specific expressed genes were detected in zws-ms and zws-217, respectively (Supplementary Table 3).

Moreover, the comparison of DEGs between two environments identified 33 DEGs that represented the same or similar expression trends (Supplementary Table 4). In other words, these genes were up- (or down-) regulated in both environments, and they were assigned as “stable DEGs,” whereas the other genes showed different expression trends, which meant zws-ms and zws-217 displayed differential expression level of those genes when grown in Xindu, but no obvious difference while grown in Ma’erkang (Supplementary Table 5). These genes were defined as “environment-specific DEGs” in this study.

### Annotations for the Differentially Expressed Genes

**FIGURE 2:**
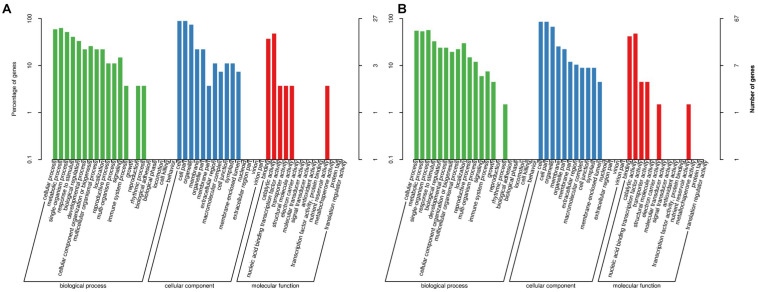
Gene Ontology (GO) terms associated with two classifications of differentially expressed genes (DEGs). **(A)** Thirty-three stable DEGs representing same or similar expression trends in both environments. **(B)** Ninety-six environment-specific DEGs showing different expression trends from Xindu to Ma’erkang. *X*-axis shows GO categories and subclasses of DEGs; *y*-axis shows number or percentage of annotated DEGs.

As mentioned earlier, we divided these DEGs into two classifications (stable and environment-specific DEGs) and then analyzed their GO annotations. GO terms were usually divided into three categories: biological processes, cellular components (CCs), and molecular functions (MFs). In classification (Figure 2A) for 33 stable DEGs, the biological process terms with the highest levels of enrichment included “metabolic process (GO:0008152)” and “cellular process (GO:0009987),” involving 17 and 16 DEGs; in the CC category, most enriched terms were “cell (GO: 0005623)” and “cell part (GO: 0044464),” whereas in the MF category, “catalytic activity (GO: 0003824)” and “binding (GO: 0005488)” accounted the top enriched terms, containing 13 and 10 DEGs, respectively. In the second classification (Figure 2B) for 96 environment-specific DEGs, the terms “single-organism process (GO:0044699)” and “cellular process (GO:0009987)” got the highest numbers of a gene, at 38 and 37, respectively; the CC and MF categories showed similar most-enriched terms to those in the first classification.

Two stable DEGs and eight environment-specific DEGs got annotations related to flower development or environment response (Tables 2, 3): on the one hand, (1) BnaC08g39130D was line-specific only in zws-ms in both environments, and it was annotated to “ovule development (GO:0048481)” and “response to cold (GO:0009409);” (2) BnaC08g42280D, associated with “vegetative to reproductive phase transition of the meristem (GO:0010228)” and “cellular response to cold (GO:0070417),” was expressed only in zws-217 in Xindu, and in Ma’erkang, it was significantly downregulated in zws-ms, which showed a similar tendency. On the other hand, as to the environment-specific DEGs, BnaA09g45320D, BnaA10g07970D, BnaAnng35580D, BnaC08g29060D, BnaC08g41780D, BnaC08g42450D, and BnaA09g44370D were downregulated in zws-ms or only expressed in zws-217 in Xindu but showed no significant difference between two lines in Ma’erkang, whereas BnaC08g40320D showed variation in Xindu only, with line-specific expression in zws-ms there. To be specific, (1) BnaA09g45320D was annotated to environment-responsive terms such as “response to heat (GO:0009408),” “response to cold (GO:0009409),” and “ovule development (GO:0048481),” its ortholog from *Arabidopsis* is *Chaperonin 10* (*CPN10*); (2) BnaA10g07970D was found a heat shock protein about “response to abiotic stimulus (GO:0009628)” and homologous to AT5G51440; (3) BnaAnng35580D was related to “response to cold (GO:0009409)” and “vegetative to the reproductive phase transition of meristem (GO:0010228).” The ortholog encoded a glycine-rich protein; (4) BnaC08g29060D was in connection with “stamen development (GO:0048443);” (5) BnaC08g41780D was relevant to “vegetative to reproductive phase transition of meristem (GO:0010228)” and “ovule development (GO:0048481).” Its ortholog, AT1G11870, encoded the seryl-tRNA synthetase and was identified as *OVA7* gene; (6) BnaC08g42450D was annotated as “stamen development (GO:0048443);” both (7) BnaA09g44370D and (8) BnaC08g40320D had a Myb-like DNA-binding domain.

**TABLE 2:** Annotations for important stable and environment-specific DEGs.

	Gene ID	GO annotation	KEGG pathway
Stable DEG	BnaC08g39130D	copper ion binding (GO:0005507); calmodulin binding (GO:0005516); ATP binding (GO:0005524); mitochondrion (GO:0005739); cytosol (GO:0005829); gluconeogenesis (GO:0006094); glycolytic process (GO:0006096); protein folding (GO:0006457); tryptophan catabolic process (GO:0006569); response to heat (GO:0009408); response to cold (GO:0009409); chloroplast thylakoid membrane (GO:0009535); chloroplast stroma (GO:0009570); response to high light intensity (GO:0009644); response to salt stress (GO:0009651); chloroplast organization (GO:0009658); indoleacetic acid biosynthetic process (GO:0009684); chloroplast envelope (GO:0009941); isopentenyl diphosphate biosynthetic process, methylerythritol 4-phosphate pathway (GO:0019288); cysteine biosynthetic process (GO:0019344); response to endoplasmic reticulum stress (GO:0034976); response to hydrogen peroxide (GO:0042542); response to cadmium ion (GO:0046686); apoplast (GO:0048046); ovule development (GO:0048481); chaperone binding (GO:0051087); positive regulation of superoxide dismutase activity (GO:1901671);	–
	BnaC08g42280D	telomere maintenance (GO:0000723); double-strand break repair via homologous recombination (GO:0000724); nucleic acid binding (GO:0003676); ATP binding (GO:0005524); nucleus (GO:0005634); DNA replication (GO:0006260); plasmodesma (GO:0009506); vegetative to reproductive phase transition of meristem (GO:0010228); ATP-dependent 3’-5’ DNA helicase activity (GO:0043140); cellular response to cold (GO:0070417); cellular response to abscisic acid stimulus (GO:0071215);	Homologous recombination (ko03440)
Environment-specific DEG	BnaA09g44370D	DNA binding (GO:0003677); chromatin binding (GO:0003682); sequence-specific DNA binding transcription factor activity (GO:0003700); nucleus (GO:0005634); regulation of transcription, DNA-templated (GO:0006355); protein targeting to membrane (GO:0006612); response to salt stress (GO:0009651); response to ethylene (GO:0009723); response to auxin (GO:0009733); response to abscisic acid (GO:0009737); response to gibberellin (GO:0009739); response to salicylic acid (GO:0009751); response to jasmonic acid (GO:0009753); positive regulation of flavonoid biosynthetic process (GO:0009963); regulation of plant-type hypersensitive response (GO:0010363); response to cadmium ion (GO:0046686);	–
	BnaA09g45320D	copper ion binding (GO:0005507); calmodulin binding (GO:0005516); ATP binding (GO:0005524); mitochondrion (GO:0005739); cytosol (GO:0005829); gluconeogenesis (GO:0006094); glycolytic process (GO:0006096); protein folding (GO:0006457); tryptophan catabolic process (GO:0006569); response to heat (GO:0009408); response to cold (GO:0009409); chloroplast thylakoid membrane (GO:0009535); chloroplast stroma (GO:0009570); response to high light intensity (GO:0009644); response to salt stress (GO:0009651); chloroplast organization (GO:0009658); indoleacetic acid biosynthetic process (GO:0009684); chloroplast envelope (GO:0009941); isopentenyl diphosphate biosynthetic process, methylerythritol 4-phosphate pathway (GO:0019288); cysteine biosynthetic process (GO:0019344); response to endoplasmic reticulum stress (GO:0034976); response to hydrogen peroxide (GO:0042542); response to cadmium ion (GO:0046686); apoplast (GO:0048046); ovule development (GO:0048481); chaperone binding (GO:0051087); positive regulation of superoxide dismutase activity (GO:1901671);	–
	BnaA10g07970D	response to stress (GO:0006950); response to abiotic stimulus (GO:0009628); cellular process (GO:0009987);	Protein processing in endoplasmic reticulum (ko04141)
	BnaAnng35580D	nucleotide binding (GO:0000166); alternative mRNA splicing, via spliceosome (GO:0000380); double-stranded DNA binding (GO:0003690); single-stranded DNA binding (GO:0003697); mRNA binding (GO:0003729); protein kinase activity (GO:0004672); nucleus (GO:0005634); mitochondrion (GO:0005739); peroxisome (GO:0005777); cytosol (GO:0005829); gluconeogenesis (GO:0006094); glycolytic process (GO:0006096); mRNA export from nucleus (GO:0006406); water transport (GO:0006833); hyperosmotic response (GO:0006972); Golgi organization (GO:0007030); response to cold (GO:0009409); response to water deprivation (GO:0009414); plasmodesma (GO:0009506); chloroplast (GO:0009507); response to salt stress (GO:0009651); etioplast organization (GO:0009662); lignin biosynthetic process (GO:0009809); response to zinc ion (GO:0010043); regulation of stomatal movement (GO:0010119); response to chitin (GO:0010200); vegetative to reproductive phase transition of meristem (GO:0010228); RNA secondary structure unwinding (GO:0010501); carotenoid biosynthetic process (GO:0016117); brassinosteroid biosynthetic process (GO:0016132); cinnamoyl-CoA reductase activity (GO:0016621); DNA duplex unwinding (GO:0032508); negative regulation of circadian rhythm (GO:0042754); protein homodimerization activity (GO:0042803); innate immune response (GO:0045087); carotenoid isomerase activity (GO:0046608); response to cadmium ion (GO:0046686); apoplast (GO:0048046); defense response to fungus (GO:0050832);	–
	BnaC08g29060D	RNA splicing, via endonucleolytic cleavage and ligation (GO:0000394); inositol hexakisphosphate binding (GO:0000822); response to molecule of bacterial origin (GO:0002237); ubiquitin-protein transferase activity (GO:0004842); nucleus (GO:0005634); vacuolar membrane (GO:0005774); methionine biosynthetic process (GO:0009086); auxin-activated signaling pathway (GO:0009734);		auxin binding (GO:0010011); stomatal complex morphogenesis (GO:0010103); pollen maturation (GO:0010152); protein ubiquitination (GO:0016567); stamen development (GO:0048443); lateral root development (GO:0048527); photoperiodism, flowering (GO:0048573); cellular response to nitrate (GO:0071249); primary root development (GO:0080022);	–
	BnaC08g40320D	chromatin binding (GO:0003682); sequence-specific DNA binding transcription factor activity (GO:0003700); nucleus (GO:0005634); regulation of transcription, DNA-templated (GO:0006355); membrane fusion (GO:0006944); identical protein binding (GO:0042802); sequence-specific DNA binding (GO:0043565); Golgi vesicle transport (GO:0048193);	–
	BnaC08g41780D	sulfur amino acid metabolic process (GO:0000096); serine-tRNA ligase activity (GO:0004828); ATP binding (GO:0005524); mitochondrion (GO:0005739); rRNA processing (GO:0006364); seryl-tRNA aminoacylation (GO:0006434); mitochondrion organization (GO:0007005); cellular amino acid biosynthetic process (GO:0008652); serine family amino acid metabolic process (GO:0009069); chloroplast (GO:0009507); embryo development ending in seed dormancy (GO:0009793); chloroplast relocation (GO:0009902); leaf morphogenesis (GO:0009965); thylakoid membrane organization (GO:0010027); photosystem II assembly (GO:0010207); vegetative to reproductive phase transition of meristem (GO:0010228); iron-sulfur cluster assembly (GO:0016226); cell differentiation (GO:0030154); regulation of protein dephosphorylation (GO:0035304); cell wall modification (GO:0042545); transcription from plastid promoter (GO:0042793); positive regulation of transcription, DNA-templated (GO:0045893); ovule development (GO:0048481);	Aminoacyl-tRNA biosynthesis (ko00970)
	BnaC08g42450D	response to molecule of bacterial origin (GO:0002237); protein serine/threonine kinase activity (GO:0004674); ATP binding (GO:0005524); plasma membrane (GO:0005886); N-terminal protein myristoylation (GO:0006499); protein targeting to membrane (GO:0006612); membrane fusion (GO:0006944); response to oxidative stress (GO:0006979); transmembrane receptor protein tyrosine kinase signaling pathway (GO:0007169); systemic acquired resistance (GO:0009627); seed germination (GO:0009845); stomatal complex morphogenesis (GO:0010103); regulation of plant-type hypersensitive response (GO:0010363); integral component of membrane (GO:0016021); negative regulation of programmed cell death (GO:0043069); protein autophosphorylation (GO:0046777); stamen development (GO:0048443); micropyle (GO:0070825);	–
	Cole_newGene_2073	intracellular membrane-bounded organelle (GO:0043231); cytoplasmic part (GO:0044444);	Protein processing in endoplasmic reticulum (ko04141); Plant-pathogen interaction (ko04626)

**TABLE 3:** Ortholog information for selected stable DEGs and nine environment-specific DEGs.

	Gene ID	Ortholog in Arabidopsis	Description
Stable DEG	BnaC08g39130D	AT1G14980	Chaperonin 10 (CPN10)
	BnaC08g42280D	AT1G10930	RECQ4A
Environment-specific DEG	BnaA09g44370D	AT1G19000	Homeodomain-like superfamily protein
	BnaA09g45320D	AT1G14980	Chaperonin 10 (CPN10)
	BnaA10g07970D	AT5G51440	HSP20-like chaperones superfamily protein
	BnaAnng35580D	AT2G21660	GLYCINE RICH PROTEIN 7 (ATGRP7)
	BnaC08g29060D	AT1G12820	Auxin signaling F-box 3 (AFB3)
	BnaC08g40320D	AT1G13450	Homeodomain-like superfamily protein
	BnaC08g41780D	AT1G11870	Seryl-tRNA synthetase (SRS), OVULE ABORTION 7 (OVA7)
	BnaC08g42450D	AT1G09970	LRR XI-23, RECEPTOR-LIKE KINASE 7 (RLK7)
	Cole_newGene_2073	AT3G07770	HEAT SHOCK PROTEIN 90.6, ATHSP90-6

**FIGURE 3:**
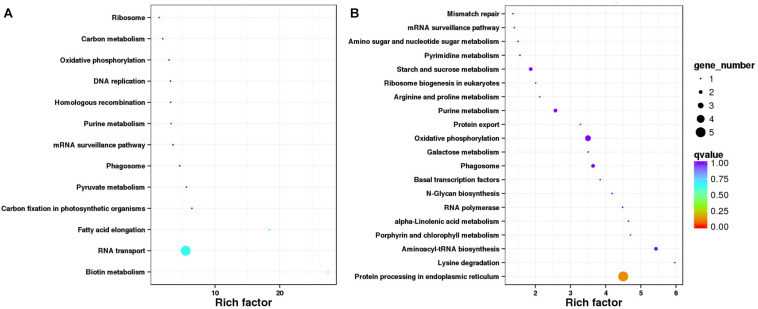
Statistics of Kyoto Encyclopedia of Genes and Genomes pathway enrichment for two classifications of differentially expressed genes (DEGs). **(A)** Data for 33 stable DEGs representing same or similar expression trends in both environments. **(B)** Data for 96 environment-specific DEGs showing different expression trends from Xindu to Ma’erkang.

KEGG pathway enrichment revealed that the stable DEG classification, the two enriched pathways (Figure 3A) with most genes, were “RNA transport (ko03013),” with BnaC08g38300D and BnaC08g40410D involved. Pathways “Biotin metabolism (ko00780)” and “Fatty acid elongation (ko00062)” got the highest enrichment factor values, at 27.4 and 18.4, and associated with BnaC01g43270D and BnaC03g65980D, respectively. As to the environment-specific DEG group (Figure 3B), “Protein processing in endoplasmic reticulum (ko04141)” got the most genes, whereas “Lysine degradation (ko00310)” with BnaA07g09660D and “Aminoacyl-tRNA biosynthesis (ko00970)” with BnaC08g41780D and a new gene got the highest values of enrichment factors.

## Discussion

Morphology of higher plants results from the interaction of genotype and environment. The multi-silique trait in rapeseed line zws-ms was found stable in Xindu for generations but absent in Ma’erkang where the climate is colder (Chai et al., 2019). In each place, there are some genes expressed differentially between zws-ms and its NIL zws-217. In Xindu, these DEGs may be the causal factors that distinguish zws-ms from zws-217 in the multi-silique trait, whereas in Ma’erkang, some of those genes turn to no significant difference in expression level between the two lines, which may be regulated by environment and switch off the multi-silique trait in zws-ms. To investigate the potential environment-regulated genes, which switch on/off the multi-silique trait, we compared DEGs generated in Xindu with those in Ma’erkang.

In our earlier report (Chai et al., 2019), we investigated the potential causal variation between zws-ms and zws-217 by the whole-genome re-sequencing, which then led to the identification of two associated regions on chromosome A09 and C08, respectively, as well as some candidate genes within them. It was based on the genomic level conferring inherent and stable genetic variations between the multi-silique and normal-silique lines, which would not be affected by environmental factors. The success of NIL construction, which conferred high genetic similarity between zws-ms and zws-217 and made them different from each other only in this multi-silique trait, ensured the accuracy of these studies. However, the expression level of transcripts can obviously change with external factors. In Chai et al. (2019), we also performed an RNA-seq and found some DEGs between zws-ms and zws-217 in Xindu, where they distinguished from each other in this multi-/single-silique trait; thus, we found some potential causal genes. However, which gene(s) switched off the multi-silique in the colder area remained unclear. Thus, in this study, we added another RNA-seq based on two lines grown in Ma’erkang, where the multi-silique disappears in zws-217, and both lines display normal siliques. By comparing the DEGs from the two environments, we can find out the changed factors.

Thus, among the 129 DEGs from Xindu, we found 33 were stable DEGs. It is worth noting that genes such as BnaA01g10540D, which was downregulated in both Xindu (log_2_FC = −4.53) and Ma’erkang (log_2_FC = −2.33), were defined as “having the same expression tendency,” whereas genes such as BnaA09g06740D, which was line-specifically expressed in zws-ms (log_2_FC = +∞) from Xindu and strongly upregulated in it (log_2_FC = 7.57) from Ma’erkang, were defined as “having similar expression tendency.” Two genes of them, BnaC08g39130D and BnaC08g42280D, got important annotations. BnaC08g39130D was line-specifically expressed in zws-ms and associated with “ovule development (GO:0048481).” Its ortholog in *Arabidopsis*, AT1G14980, encodes chaperonin 10, and this type of protein can be involved in physiological processes such as plant seed abortion (Hanania et al., 2007). BnaC08g42280D was strongly downregulated in zws-ms and annotated to “vegetative to reproductive phase transition of meristem (GO:0010228).” This implies that BnaC08g42280D probably serves as an inhibitor of the formation of multi-silique. Its ortholog, AT1G10930, is RECQ4A. Its mutant is hypersensitive to ultraviolet light and sensitive to methyl methanesulfonate. The two genes confer intrinsic and stable variations in zws-ms and are not influenced by the environment.

Aiming to investigate the thermomorphogenesis-relative genes, we then turned to those environment-specific DEGs, which were defined as those genes such as BnaA05g21710D, which showed significant upregulation in Xindu (log_2_FC = 3.12) but no obvious change in Ma’erkang (log_2_FC = 0.93), that were considered as “environment-specific DEGs” herein. Among the 96 environment-specific DEGs, nine were noteworthy. BnaA09g45320D shared the same ortholog (AT1G14980) with BnaC08g39130D, but unlike the latter, it was only expressed in zws-217 in Xindu and showed no significant difference in Ma’erkang. Its annotation may explain this in response to heat (GO:0009408) and response to cold (GO:0009409). Moreover, it was also annotated to “ovule development (GO:0048481).” Take chaperonin 21 as an example: it was found differentially expressed in seedless and seeded grapes; its silencing resulted in seed abortion in transgenic tobacco (*Nicotiana benthamiana*) and seedless fruits in transgenic tomato (*Lycopersicum esculentum*) (Hanania et al., 2007). In Xindu, BnaA09g45320D was line-specific in the normal line, whereas in Ma’erkang, zws-ms and zws-217 showed no significant difference in it. This implied a potential relevance with the multi-silique. The ortholog of BnaC08g41780D, AT1G11870, encodes a seryl-tRNA synthetase, i.e., OVA7, of which disruption can result in ovule abortion in *Arabidopsis* (Berg et al., 2005). When zws-ms represents multi-silique in Xindu, the expression of BnaC08g41780D was not detected in it, which indicates a possibility of it modifying the trait in a warm area. Thus, these two genes, due to the consistency of their expression verities with the changes of environment, as well as their annotations, are considered the most important thermomorphogenesis-related genes of all the candidates.

Although the other environmental-specific DEGs also implicated some indirect clues: BnaA09g44370D and BnaC08g40320D were annotated to MYB-like or MYB/SANT-like DNA-binding domain; and their orthologs, AT1G19000 and AT1G13450, were both homeodomain-like superfamily proteins. Some MYB proteins were found regulated by phytochrome-interacting factor 4, an important thermomorphogenesis factor in *Arabidopsis* (Wang et al., 2018). In addition to flower color, MYB transcription factors are also involved in the regulation of flower development: transgenic tobacco overexpressing MdMYB3 from apple (Malus × domestica) got longer peduncles of flowers and styles of pistils (Vimolmangkang et al., 2013). BnaA10g07970D and Cole_newGene_2073 were identified as heat shock proteins, which respond to temperature and are involved in signal transduction, protein trafficking, protein degradation, maintaining protein homeostasis, and so on (Raman and Suguna, 2015), but whether/how they are related to flower/fruit shape needs further research. AT2G21660 is homologous to BnaAnng35580D. It is *ATGRP7* and encodes a small glycine-rich RNA binding protein. Loss-of-function mutations are late flowering in a non-photoperiodic manner. BnaC08g29060D was annotated to “photoperiodism, flowering (GO: 0048573)” and identified as homologous to AT1G12820, which is auxin signaling F-box 3 and involved in primary and lateral root growth inhibition (Dong et al., 2006). AT1G09970 is an ortholog of BnaC08g42450D and identified as *RECEPTOR-LIKE KINASE 7*. According to the existing knowledge, it is involved in the control of germination speed and oxidant stress tolerance.

There is another sort of gene, which was not discussed. Although these genes got annotations regarding environment-response, they showed no difference resulting from environmental changes. Take BnaC08g39990D for example; it was annotated to “response to cold (GO:0009409),” which seemed potentially related to what we were seeking. However, further analysis indicated that it was always upregulated in zws-ms in both Xindu and Ma’erkang, showing no variety as the environment changed. Thus, its expression level had a low correlation with environmental factors acting on the switching on/off of the multi-silique formation. So, this kind of gene was not emphasized herein.

Rapeseed is not the first crop conferring the multi-pistil trait reported. In fact, wheat contributes relatively sufficient studies about it. Researchers reported their multi-pistil wheat materials, in which the trait can be controlled by a recessive gene, two recessive non-complementary genes, or a single dominant gene, and the loci were located in different chromosomes (Zhu et al., 2019). Even so, no underlying gene in wheat has not been cloned so far (Zhu et al., 2019).

Notably, the multi-pistil trait was also reported in sweet cherry (*P. avium* L.), co-regulated by genes *PaMADS3/4/5*, members of the MADS-box family (Liu et al., 2019; Wang et al., 2019). Interestingly, that was also induced by temperature: it occurred more frequently in the warm region of Shanghai than in the cool region of Dalian. This coincides with our discovery from rapeseed. Notably, there are many environmental factors, but the temperature is the most significant one, varying from Xindu to Ma’erkang. Although some other factors, such as photoperiod, are also vital for plant development, there has been no report showing it can change pistil number in flower; it is known to regulate flowering time, as well as some physiological characters such as hypocotyl length, cotyledon angle, chlorophyll content, etc., so far. Instead, a change in temperature, as mentioned earlier (Liu et al., 2019; Wang et al., 2019), can promote the multi-pistil formation (in sweet cherry). Thus, we suppose it is the temperature that would switch on/off the multi-silique herein.

The multi-silique phenotype means different things to different plants. For some crops, the multi-silique phenotype was considered as an advantage: in wheat, for example, it is supposed to have the potential to increase yields (Wei, 2017; Zhu et al., 2019); on the other hand, for some other plants, such as sweet cherry, the multi-pistil is regarded as a disadvantage physiological disorder decreasing its commercial value (Liu et al., 2019). As to rapeseed, although how to use the multi-silique line to increase the seed yield, as well as the benefit of commercial use, still needs more investigations. We should explain that the mountain area (cold region such as Ma’erkang) is not the major rapeseed-producing area in Sichuan Province; we grow rapeseed there mainly for scientific purposes: the shuttle breeding makes it possible to produce two generations in 1 year, accelerating the population construction processes. Collectively, this study at least provides a new perspective into flower/fruit development in higher plants. In the next stage, the functional validation of these candidate genes will be carried out by investigating the phenotypic, biochemical, and molecular changes in the transgenic plants (both overexpression and knockdown).

## Conclusion

By comparing DEGs between zws-ms and zws-217 in Xindu with those in Ma’erkang and referring to the gene annotations, we selected BnaA09g45320D (*chaperonin 10*-homologous) and BnaC08g41780D (*OVA7*-homologous) as the possible thermomorphogenesis-related genes, switching on/off the multi-silique under different environments.

## Data Availability Statement

The datasets presented in this study can be found in online repositories. The names of the repository/repositories and accession number(s) can be found below: NCBI and accession number PRJNA736189.

## Author Contributions

LC and LJ conceived the experiment. LC and JZ performed the research. HL, CC, JJ, and BZ contributed to data analysis. LC wrote the manuscript. LJ and LW reviewed and revised the manuscript. All authors reviewed and approved this submission.

## Conflict of Interest

The authors declare that the research was conducted in the absence of any commercial or financial relationships that could be construed as a potential conflict of interest.

## Publisher’s Note

All claims expressed in this article are solely those of the authors and do not necessarily represent those of their affiliated organizations, or those of the publisher, the editors and the reviewers. Any product that may be evaluated in this article, or claim that may be made by its manufacturer, is not guaranteed or endorsed by the publisher.

## Footnotes

1 www.genoscope.cns.fr/brassicanapus/data/

2 http://geneontology.org/

3 http://www.genome.jp/kegg/

4 www.arabidopsis.org/

## Supplementary Material

Supplementary Table 1 The 117 DEGs between zws-ms and zws-217 from colder area Ma’erkang.

Supplementary Table 2 Line-specific expressed genes from Xindu.

Supplementary Table 3 Line-specific expressed genes from Ma’erkang.

Supplementary Table 4 The 33 stable DEGs with the same or similar expression tendency in two environments.

Supplementary Table 5 The 96 environment-specific DEGs with different expression tendency in two environments.

## References

[B1] Benjamini Y. Hochberg Y. (1995). Controlling the False Discovery Rate: A Practical and Powerful Approach to Multiple Testing. *J. Roy. Stat. Soc. B* 57 289–300. 10.1111/j.2517-6161.1995.tb02031.x

[B2] Berg M. Rogers R. Muralla R. Meinke D. (2005). Requirement of aminoacyl-tRNA synthetases for gametogenesis and embryo development in Arabidopsis. *Plant J.* 44 866–878. 10.1111/j.1365-313X.2005.02580.x 16297076

[B3] Casal J. J. Balasubramanian S. (2019). Thermomorphogenesis. *Annu. Rev. Plant Biol.* 70 321–346. 10.1146/annurev-arplant-050718-095919 30786235

[B4] Chai L. Zhang J. Li H. Jiang J. Cui C. Zheng B. (2020b). Dynamic Transcriptome Analysis of A Multi-Silique Trait in Rapeseed (*Brassica napus* L.). *Int. J. Agric. Biol.* 24 1625–1632.

[B5] Chai L. Zhang J. Li H. Zheng B. Jiang J. Cui C. (2020a). Investigation for a multi-silique trait in *Brassica napus* by alternative splicing analysis. *PeerJ.* 8:e10135. 10.7717/peerj.10135 33083151 PMC7548069

[B6] Chai L. Zhang J. Lu K. Li H. Wu L. Wan H. (2019). Identification of genomic regions associated with multi-silique trait in *Brassica napus*. *BMC Genomics* 20:304. 10.1186/s12864-019-5675-4 31014236 PMC6480887

[B7] Ding Y. Shi Y. Yang S. (2020). Molecular Regulation of Plant Responses to Environmental Temperatures. *Mol. Plant* 13 544–564. 10.1016/j.molp.2020.02.004 32068158

[B8] Dong L. Wang L. Zhang Y. E. Zhang Y. Deng X. Xue Y. (2006). An Auxin-Inducible F-Box Protein CEGENDUO Negatively Regulates Auxin-Mediated Lateral Root Formation in *Arabidopsis*. *Plant Mol. Biol.* 60 599–615. 10.1007/s11103-005-5257-5 16525894

[B9] Duan Z. Shen C. Li Q. Lü G. Ni Y. Yu D. (2015). Identification of a novel male sterile wheat mutant *dms* conferring dwarf status and multi-pistils. *J. Integr. Agr.* 14 1706–1714. 10.1016/S2095-3119(14)60936-9

[B10] Friend D. J. C. (1965). TILLERING AND LEAF PRODUCTION IN WHEAT AS AFFECTED BY TEMPERATURE AND LIGHT INTENSITY. *Can. J. Bot.* 43 1063–1076. 10.1139/b65-123 33356898

[B11] Geng X. Jiang C. Yang J. Wang L. Wu X. Wei W. (2016). Rapid Identification of Candidate Genes for Seed Weight Using the SLAF-Seq Method in *Brassica napus*. *PLoS One* 11:e147580. 10.1371/journal.pone.0147580 26824525 PMC4732658

[B12] Guo J. Zhang G. Song Y. Li Z. Ma S. Niu N. (2019). Comparative proteomic analysis of multi-ovary wheat under heterogeneous cytoplasm suppression. *BMC Plant Biol.* 19:175. 10.1186/s12870-019-1778-y 31046676 PMC6498644

[B13] Hanania U. Velcheva M. Or E. Flaishman M. Sahar N. Perl A. (2007). Silencing of chaperonin 21, that was differentially expressed in inflorescence of seedless and seeded grapes, promoted seed abortion in tobacco and tomato fruits. *Transgenic Res.* 16 515–525. 10.1007/s11248-006-9044-0 17103240

[B14] Ibañez C. Poeschl Y. Peterson T. Bellstädt J. Denk K. Gogol-Döring A. (2017). Ambient temperature and genotype differentially affect developmental and phenotypic plasticity in Arabidopsis thaliana. *BMC Plant Biol.* 17:114. 10.1186/s12870-017-1068-5 28683779 PMC5501000

[B15] Kanehisa M. Araki M. Goto S. Hattori M. Hirakawa M. Itoh M. (2007). KEGG for linking genomes to life and the environment. *Nucleic Acids Res.* 36 D480–D484. 10.1093/nar/gkm882 18077471 PMC2238879

[B16] Kim D. Pertea G. Trapnell C. Pimentel H. Kelley R. Salzberg S. L. (2013). TopHat2: accurate alignment of transcriptomes in the presence of insertions, deletions and gene fusions. *Genome Biol.* 14:R36. 10.1186/gb-2013-14-4-r36 23618408 PMC4053844

[B17] Lee Y. Kim K. Lee J. Cha Y. Moon Y. Song Y. (2018). Comprehensive Transcriptome Profiling in Relation to Seed Storage Compounds in Tetralocular *Brassica rapa*. *J. Plant Growth Regul.* 37 867–882. 10.1007/s00344-018-9784-0

[B18] Liu J. Wang J. She W. Wang L. Luo M. Chen Y. (2019). MADS-Box Genes are Involved in Cultivar- and Temperature-Dependent Formation of Multi-pistil and Polycarpy in *Prunus avium* L. *J. Plant Growth Regul.* 38 1017–1027. 10.1007/s00344-018-09911-8

[B19] Love M. I. Huber W. Anders S. (2014). Moderated estimation of fold change and dispersion for RNA-seq data with DESeq2. *Genome Biol.* 15:550. 10.1186/s13059-014-0550-8 25516281 PMC4302049

[B20] Mao X. Cai T. Olyarchuk J. G. Wei L. (2005). Automated genome annotation and pathway identification using the KEGG Orthology (KO) as a controlled vocabulary. *Bioinformatics* 21 3787–3793. 10.1093/bioinformatics/bti430 15817693

[B21] Nair R. M. Peck D. M. Dundas I. S. Samac D. A. Moore A. Randles J. W. (2008). Morphological characterisation and genetic analysis of a bi-pistil mutant (*bip*) in *Medicago truncatula* Gaertn. *Sex. Plant Reprod.* 21 133–141. 10.1007/s00497-008-0073-0

[B22] Raman S. Suguna K. (2015). Functional characterization of heat−shock protein 90 from *Oryza sativa* and crystal structure of its N−terminal domain. *Acta Crystallogr. Sect. F: Struct. Biol. Commun.* 71 688–696. 10.1107/S2053230X15006639 26057797 PMC4461332

[B23] Shen W. Qin P. Yan M. Li B. Wu Z. Wen J. (2019). Fine mapping of a silique length- and seed weight-related gene in *Brassica napus*. *Theor. Appl. Genet.* 132 2985–2996. 10.1007/s00122-019-03400-6 31321475

[B24] Sionit N. Strain B. R. Flint E. P. (1987). Interaction of temperature and co_2_ enrichment on soybean: growth and dry matter partitioning. *Can. J. Plant Sci.* 67 59–67. 10.4141/cjps87-007

[B25] Vimolmangkang S. Han Y. Wei G. Korban S. S. (2013). An apple MYB transcription factor, *MdMYB3*, is involved in regulation of anthocyanin biosynthesis and flower development. *BMC Plant Biol.* 13:176. 10.1186/1471-2229-13-176 24199943 PMC3833268

[B26] Wang J. Liu J. Jiu S. Li Y. Whiting M. She W. (2019). The MADS-box genes *PaMADS3/4/5* co-regulate multi-pistil formation induced by high temperature in *Prunus avium* L. *Sci. Hortic.-Amsterdam* 256:108593. 10.1016/j.scienta.2019.108593

[B27] Wang S. Ding L. Liu J. X. Han J. J. (2018). PIF4-Regulated Thermo-responsive Genes in *Arabidopsis*. *Biotechnol. Bull.* 34 57–65.

[B28] Wang X. Chen L. Wang A. Wang H. Tian J. Zhao X. (2016). Quantitative trait loci analysis and genome-wide comparison for silique related traits in *Brassica napus*. *BMC Plant Biol.* 16:71. 10.1186/s12870-016-0759-7 27000872 PMC4802616

[B29] Wei S. H. (2017). Characterization and expression of *WAG-2* transcripts in a wheat three-pistil mutant line. *Russ. J. Plant Physl.* 64 680–687. 10.1134/S1021443717050156

[B30] Xiao L. Zhao H. Zhao Z. Du D. Xu L. Yao Y. (2013). Genetic and physical fine mapping of a multilocular gene *Bjln1* in *Brassica juncea* to a 208-kb region. *Mol. Breeding* 32 373–383. 10.1007/s11032-013-9877-1

[B31] Xu P. Lv Z. Zhang X. Wang X. Pu Y. Wang H. (2014). Identification of molecular markers linked to trilocular gene (*mc1*) in *Brassica juncea* L. *Mol. Breeding* 33 425–434. 10.1007/s11032-013-9960-7

[B32] Yadava S. K. Paritosh K. Panjabi-Massand P. Gupta V. Chandra A. Sodhi Y. S. (2014). Tetralocular ovary and high silique width in yellow sarson lines of *Brassica rapa* (subspecies *trilocularis*) are due to a mutation in Bra034340 gene, a homologue of *CLAVATA3* in Arabidopsis. *Theor. Appl. Genet.* 127 2359–2369. 10.1007/s00122-014-2382-z 25205130

[B33] Yang P. Shu C. Chen L. Xu J. Wu J. Liu K. (2012). Identification of a major QTL for silique length and seed weight in oilseed rape (*Brassica napus* L.). *Theor. Appl. Genet.* 125 285–296. 10.1007/s00122-012-1833-7 22406980

[B34] Yang Z. Chen Z. Peng Z. Yu Y. Liao M. Wei S. (2017). Development of a high-density linkage map and mapping of the three-pistil gene (*Pis1*) in wheat using GBS markers. *BMC Genomics* 18:567. 10.1186/s12864-017-3960-7 28760136 PMC5537994

[B35] Yang Z. Peng Z. Yang H. Yang J. Wei S. Cai P. (2011). Suppression Subtractive Hybridization Identified Differentially Expressed Genes in Pistil Mutations in Wheat. *Plant Mol. Biol. Rep.* 29 431–439. 10.1007/s11105-010-0249-2

[B36] Young M. D. Wakefield M. J. Smyth G. K. Oshlack A. (2010). Gene ontology analysis for RNA-seq: accounting for selection bias. *Genome Biol.* 11:R14. 10.1186/gb-2010-11-2-r14 20132535 PMC2872874

[B37] Yu Z. Y. Luo Q. Peng Z. S. Wei S. H. Yang Z. J. Yamamoto N. (2020). Genetic mapping of the three-pistil gene *Pis1* in an F_2_ population derived from a synthetic hexaploid wheat using multiple molecular marker systems. *Cereal Res. Commun.* 2020:078. 10.1007/s42976-020-00078-1

[B38] Zheng H. Zhang J. Zhuang H. Zeng X. Tang J. Wang H. (2019). Gene mapping and candidate gene analysis of multi-floret spikelet 3 (*mfs3*) in rice (*Oryza sativa* L.). *J. Integr. Agr.* 18 2673–2681. 10.1016/S2095-3119(19)62652-3

[B39] Zhu X. Ni Y. He R. Jiang Y. Li Q. Niu J. (2019). Genetic mapping and expressivity of a wheat multi-pistil gene in mutant *12TP*. *J. Integr. Agr.* 18 532–538. 10.1016/S2095-3119(18)61935-5

