# Supplementary material for: Investigation of Thermomorphogenesis-Related Genes for a Multi-Silique Trait in Brassica napus by Comparative Transcriptome Analysis
Source: Front Genet. 2021 Jul 23;12:678804. doi: 10.3389/fgene.2021.678804 (PMC8343136; doi:10.3389/fgene.2021.678804)
Supplement: Supplementary Table 1 — The 117 DEGs between zws-ms and zws-217 from colder area Ma’erkang. [file Table_1.DOCX]

**Supplementary Table 1|** The 117 DEGs between zws-ms and zws-217 from colder area Ma’erkang.

| Gene ID | FDR | log_2_FC | regulated | GO annotation | KEGG pathway annotation |
| --- | --- | --- | --- | --- | --- |
| BnaA01g10540D | 0.000283 | -2.32916 | down | Cellular Component: nucleus (GO:0005634); | DNA replication (ko03030) |
| BnaA01g22780D | 1.07E-20 | -∞ | down | Molecular Function: transcription coactivator activity (GO:0003713); Cellular Component: cytoplasm (GO:0005737); Molecular Function: zinc ion binding (GO:0008270); | -- |
| BnaA01g26600D | 2.98E-09 | -3.12625 | down | Cellular Component: nucleus (GO:0005634); Cellular Component: Golgi apparatus (GO:0005794); Biological Process: starch metabolic process (GO:0005982); Biological Process: plant-type secondary cell wall biogenesis (GO:0009834); Biological Process: glucuronoxylan biosynthetic process (GO:0010417); Molecular Function: glucuronoxylan glucuronosyltransferase activity (GO:0080116); | -- |
| BnaA01g36650D | 9.06E-06 | 2.480704 | up | Molecular Function: sugar:proton symporter activity (GO:0005351); Biological Process: purine nucleobase transport (GO:0006863); Cellular Component: integral component of membrane (GO:0016021); Biological Process: carbohydrate transmembrane transport (GO:0034219); | -- |
| BnaA02g02700D | 6.92E-10 | -3.73711 | down | Cellular Component: vacuolar membrane (GO:0005774); Cellular Component: plasma membrane (GO:0005886); Biological Process: plant-type secondary cell wall biogenesis (GO:0009834); Biological Process: xylem development (GO:0010089); Biological Process: cellulose microfibril organization (GO:0010215); Biological Process: glucuronoxylan metabolic process (GO:0010413); Biological Process: cell growth (GO:0016049); Cellular Component: anchored component of membrane (GO:0031225); Biological Process: xylan biosynthetic process (GO:0045492); | -- |
| BnaA02g03580D | 0.000677 | -2.57738 | down | Cellular Component: plasma membrane (GO:0005886); Molecular Function: zinc ion binding (GO:0008270); Cellular Component: chloroplast (GO:0009507); Biological Process: plant-type secondary cell wall biogenesis (GO:0009834); Biological Process: xylem development (GO:0010089); Biological Process: vegetative to reproductive phase transition of meristem (GO:0010228); Biological Process: rhamnogalacturonan I side chain metabolic process (GO:0010400); Biological Process: glucuronoxylan metabolic process (GO:0010413); Cellular Component: integral component of membrane (GO:0016021); Molecular Function: cellulose synthase (UDP-forming) activity (GO:0016760); Biological Process: protein desumoylation (GO:0016926); Biological Process: cellulose biosynthetic process (GO:0030244); Biological Process: xylan biosynthetic process (GO:0045492); Biological Process: hydrogen peroxide biosynthetic process (GO:0050665); Biological Process: cell wall thickening (GO:0052386); | -- |
| BnaA03g35870D | 0.000399 | 2.542677 | up | Molecular Function: guanylate kinase activity (GO:0004385); Cellular Component: cytoplasm (GO:0005737); Biological Process: purine nucleotide metabolic process (GO:0006163); Biological Process: response to chitin (GO:0010200); Biological Process: phosphorylation (GO:0016310); Biological Process: endoplasmic reticulum unfolded protein response (GO:0030968); Biological Process: defense response to fungus (GO:0050832); | Purine metabolism (ko00230) |
| BnaA03g58390D | 3.08E-07 | -2.7003 | down | Cellular Component: cell wall (GO:0005618); Cellular Component: plasma membrane (GO:0005886); Biological Process: response to osmotic stress (GO:0006970); Biological Process: response to water deprivation (GO:0009414); Biological Process: plant-type secondary cell wall biogenesis (GO:0009834); Biological Process: salicylic acid mediated signaling pathway (GO:0009863); Biological Process: jasmonic acid mediated signaling pathway (GO:0009867); Biological Process: ethylene-activated signaling pathway (GO:0009873); Biological Process: positive regulation of abscisic acid biosynthetic process (GO:0010116); Biological Process: glucuronoxylan metabolic process (GO:0010413); Cellular Component: integral component of membrane (GO:0016021); Molecular Function: cellulose synthase (UDP-forming) activity (GO:0016760); Biological Process: cellulose biosynthetic process (GO:0030244); Biological Process: defense response to bacterium (GO:0042742); Biological Process: xylan biosynthetic process (GO:0045492); Biological Process: defense response to fungus (GO:0050832); Biological Process: cell wall thickening (GO:0052386); | -- |
| BnaA05g07340D | 7.30E-07 | -3.69898 | down | Cellular Component: Golgi apparatus (GO:0005794); Biological Process: plant-type secondary cell wall biogenesis (GO:0009834); Biological Process: glucuronoxylan biosynthetic process (GO:0010417); Molecular Function: galactosylgalactosylxylosylprotein 3-beta-glucuronosyltransferase activity (GO:0015018); Cellular Component: membrane (GO:0016020); Molecular Function: xylosyltransferase activity (GO:0042285); | -- |
| BnaA05g23800D | 0.000106 | 2.330656 | up | Cellular Component: nucleus (GO:0005634); | -- |
| BnaA05g24130D | 5.53E-06 | -2.19901 | down | -- | -- |
| BnaA06g32330D | 3.30E-16 | -3.60636 | down | Molecular Function: copper ion binding (GO:0005507); Cellular Component: plasma membrane (GO:0005886); Molecular Function: electron carrier activity (GO:0009055); Biological Process: glucuronoxylan metabolic process (GO:0010413); Cellular Component: anchored component of membrane (GO:0031225); Biological Process: xylan biosynthetic process (GO:0045492); | -- |
| BnaA06g36370D | 1.44E-08 | -2.43942 | down | Cellular Component: plasma membrane (GO:0005886); Molecular Function: zinc ion binding (GO:0008270); Biological Process: plant-type secondary cell wall biogenesis (GO:0009834); Biological Process: salicylic acid mediated signaling pathway (GO:0009863); Biological Process: jasmonic acid mediated signaling pathway (GO:0009867); Biological Process: ethylene-activated signaling pathway (GO:0009873); Biological Process: glucuronoxylan metabolic process (GO:0010413); Cellular Component: integral component of membrane (GO:0016021); Molecular Function: cellulose synthase (UDP-forming) activity (GO:0016760); Biological Process: cellulose biosynthetic process (GO:0030244); Biological Process: defense response to bacterium (GO:0042742); Biological Process: xylan biosynthetic process (GO:0045492); Biological Process: defense response to fungus (GO:0050832); Biological Process: cell wall thickening (GO:0052386); | -- |
| BnaA07g02290D | 0.000449 | 2.522251 | up | Cellular Component: mitochondrion (GO:0005739); Biological Process: protein glycosylation (GO:0006486); Biological Process: transport (GO:0006810); Cellular Component: integral component of membrane (GO:0016021); | -- |
| BnaA07g04500D | 8.90E-07 | 3.568468 | up | Cellular Component: mitochondrion (GO:0005739); | -- |
| BnaA07g19340D | 9.04E-09 | -4.41001 | down | Cellular Component: endoplasmic reticulum lumen (GO:0005788); Cellular Component: plasma membrane (GO:0005886); Biological Process: protein folding (GO:0006457); Biological Process: ER-nucleus signaling pathway (GO:0006984); Biological Process: response to heat (GO:0009408); Biological Process: systemic acquired resistance (GO:0009627); Biological Process: response to high light intensity (GO:0009644); Molecular Function: heat shock protein binding (GO:0031072); Biological Process: response to endoplasmic reticulum stress (GO:0034976); Biological Process: response to hydrogen peroxide (GO:0042542); Molecular Function: unfolded protein binding (GO:0051082); Biological Process: pathogen-associated molecular pattern dependent induction by symbiont of host innate immune response (GO:0052033); | Protein processing in endoplasmic reticulum (ko04141) |
| BnaA08g07600D | 9.35E-05 | -4.24524 | down | Molecular Function: hydrolase activity, hydrolyzing O-glycosyl compounds (GO:0004553); Cellular Component: plasma membrane (GO:0005886); Biological Process: carbohydrate metabolic process (GO:0005975); Biological Process: embryo development ending in seed dormancy (GO:0009793); Biological Process: pollen exine formation (GO:0010584); Molecular Function: cation binding (GO:0043169); | -- |
| BnaA08g29520D | 0.000497 | 2.241575 | up | Biological Process: carbohydrate metabolic process (GO:0005975); Biological Process: cellular metabolic process (GO:0044237); | -- |
| BnaA09g06740D | 1.81E-17 | 7.570671 | up | Cellular Component: nucleus (GO:0005634); | -- |
| BnaA09g15210D | 2.02E-09 | 2.250633 | up | Molecular Function: carboxylic ester hydrolase activity (GO:0004091); Cellular Component: extracellular region (GO:0005576); Cellular Component: mitochondrion (GO:0005739); Biological Process: intra-Golgi vesicle-mediated transport (GO:0006891); Biological Process: membrane fusion (GO:0006944); Cellular Component: integral component of membrane (GO:0016021); Molecular Function: transferase activity, transferring acyl groups (GO:0016746); | -- |
| BnaA09g17560D | 0.000551 | -2.39996 | down | Molecular Function: iron ion binding (GO:0005506); Cellular Component: extracellular region (GO:0005576); Molecular Function: electron carrier activity (GO:0009055); Biological Process: response to water deprivation (GO:0009414); Biological Process: response to red or far red light (GO:0009639); Biological Process: response to chitin (GO:0010200); Molecular Function: (+)-abscisic acid 8'-hydroxylase activity (GO:0010295); Molecular Function: oxygen binding (GO:0019825); Molecular Function: heme binding (GO:0020037); Biological Process: abscisic acid catabolic process (GO:0046345); Biological Process: release of seed from dormancy (GO:0048838); Biological Process: defense response to fungus (GO:0050832); Biological Process: oxidation-reduction process (GO:0055114); | Carotenoid biosynthesis (ko00906) |
| BnaA09g23510D | 1.24E-13 | -2.98332 | down | Cellular Component: extracellular region (GO:0005576); Cellular Component: mitochondrion (GO:0005739); Cellular Component: Golgi apparatus (GO:0005794); Biological Process: lignin metabolic process (GO:0009808); Biological Process: trichoblast differentiation (GO:0010054); Biological Process: glucuronoxylan metabolic process (GO:0010413); Molecular Function: glucuronoxylan 4-O-methyltransferase activity (GO:0030775); Biological Process: xylan biosynthetic process (GO:0045492); | -- |
| BnaA09g26320D | 4.16E-05 | 2.219919 | up | Molecular Function: transcription factor activity, sequence-specific DNA binding (GO:0003700); Biological Process: regulation of transcription, DNA-templated (GO:0006355); Molecular Function: sequence-specific DNA binding (GO:0043565); | -- |
| BnaA09g27450D | 8.11E-06 | -2.34559 | down | Molecular Function: purine nucleobase transmembrane transporter activity (GO:0005345); Cellular Component: integral component of plasma membrane (GO:0005887); Biological Process: purine nucleobase transport (GO:0006863); Cellular Component: chloroplast (GO:0009507); Biological Process: cytokinin transport (GO:0010184); Molecular Function: purine nucleoside transmembrane transporter activity (GO:0015211); Biological Process: purine nucleoside transmembrane transport (GO:0015860); Biological Process: cellular response to phosphate starvation (GO:0016036); | -- |
| BnaA09g48320D | 5.25E-07 | -2.64282 | down | Molecular Function: structural constituent of ribosome (GO:0003735); Cellular Component: nucleolus (GO:0005730); Biological Process: translation (GO:0006412); Cellular Component: chloroplast (GO:0009507); Cellular Component: cytosolic large ribosomal subunit (GO:0022625); | Ribosome (ko03010) |
| BnaA10g10690D | 7.40E-10 | +∞ | up | Molecular Function: ATP transmembrane transporter activity (GO:0005347); Cellular Component: mitochondrial inner membrane (GO:0005743); Biological Process: mitochondrial transport (GO:0006839); Biological Process: anther development (GO:0048653); | -- |
| BnaAnng13970D | 1.42E-13 | -3.28532 | down | Molecular Function: copper ion binding (GO:0005507); Molecular Function: L-ascorbate oxidase activity (GO:0008447); Biological Process: plant-type secondary cell wall biogenesis (GO:0009834); Biological Process: glucuronoxylan metabolic process (GO:0010413); Biological Process: xylan biosynthetic process (GO:0045492); Biological Process: lignin catabolic process (GO:0046274); Cellular Component: apoplast (GO:0048046); Molecular Function: hydroquinone:oxygen oxidoreductase activity (GO:0052716); Biological Process: oxidation-reduction process (GO:0055114); | -- |
| BnaAnng17190D | 2.15E-18 | +∞ | up | Biological Process: maltose metabolic process (GO:0000023); Biological Process: sucrose metabolic process (GO:0005985); Biological Process: fructose metabolic process (GO:0006000); Biological Process: pentose-phosphate shunt (GO:0006098); Biological Process: rRNA processing (GO:0006364); Biological Process: response to cold (GO:0009409); Cellular Component: chloroplast stroma (GO:0009570); Biological Process: response to blue light (GO:0009637); Biological Process: photosynthetic electron transport in photosystem I (GO:0009773); Biological Process: chloroplast relocation (GO:0009902); Biological Process: thylakoid membrane organization (GO:0010027); Biological Process: response to red light (GO:0010114); Biological Process: photosystem II assembly (GO:0010207); Biological Process: response to far red light (GO:0010218); Cellular Component: stromule (GO:0010319); Biological Process: chlorophyll biosynthetic process (GO:0015995); Biological Process: carotenoid biosynthetic process (GO:0016117); Biological Process: starch biosynthetic process (GO:0019252); Biological Process: reductive pentose-phosphate cycle (GO:0019253); Biological Process: cellular cation homeostasis (GO:0030003); Biological Process: fructose 1,6-bisphosphate metabolic process (GO:0030388); Biological Process: regulation of protein dephosphorylation (GO:0035304); Molecular Function: fructose 1,6-bisphosphate 1-phosphatase activity (GO:0042132); Biological Process: defense response to bacterium (GO:0042742); Biological Process: positive regulation of catalytic activity (GO:0043085); Molecular Function: metal ion binding (GO:0046872); Cellular Component: apoplast (GO:0048046); Biological Process: divalent metal ion transport (GO:0070838); | Glycolysis / Gluconeogenesis (ko00010); Pentose phosphate pathway (ko00030); Fructose and mannose metabolism (ko00051); Carbon fixation in photosynthetic organisms (ko00710); Carbon metabolism (ko01200) |
| BnaAnng17230D | 5.27E-12 | 3.298714 | up | Cellular Component: extracellular region (GO:0005576); Cellular Component: vacuole (GO:0005773); | -- |
| BnaAnng18460D | 5.57E-11 | -3.58458 | down | Cellular Component: plasma membrane (GO:0005886); Biological Process: plant-type secondary cell wall biogenesis (GO:0009834); Cellular Component: anchored component of membrane (GO:0031225); | -- |
| BnaAnng19640D | 5.03E-13 | +∞ | up | -- | -- |
| BnaAnng30260D | 5.27E-07 | 3.71287 | up | Molecular Function: transcription factor activity, sequence-specific DNA binding (GO:0003700); Cellular Component: nucleus (GO:0005634); Biological Process: response to xenobiotic stimulus (GO:0009410); Biological Process: response to ethylene (GO:0009723); Biological Process: hormone-mediated signaling pathway (GO:0009755); Biological Process: endoplasmic reticulum unfolded protein response (GO:0030968); Biological Process: positive regulation of transcription, DNA-templated (GO:0045893); Molecular Function: protein heterodimerization activity (GO:0046982); Biological Process: positive regulation of seed maturation (GO:2000693); | -- |
| BnaC01g02500D | 2.05E-07 | 5.877495 | up | -- | -- |
| BnaC01g04850D | 5.47E-06 | -3.43102 | down | Cellular Component: Golgi apparatus (GO:0005794); Biological Process: starch metabolic process (GO:0005982); Biological Process: plant-type secondary cell wall biogenesis (GO:0009834); Biological Process: meristem initiation (GO:0010014); Biological Process: xylem development (GO:0010089); Biological Process: glucuronoxylan biosynthetic process (GO:0010417); Molecular Function: glucuronoxylan glucuronosyltransferase activity (GO:0080116); | -- |
| BnaC01g43270D | 2.84E-05 | 4.066506 | up | Molecular Function: magnesium ion binding (GO:0000287); Molecular Function: adenosylmethionine-8-amino-7-oxononanoate transaminase activity (GO:0004015); Molecular Function: dethiobiotin synthase activity (GO:0004141); Molecular Function: ATP binding (GO:0005524); Cellular Component: mitochondrion (GO:0005739); Biological Process: DNA replication initiation (GO:0006270); Biological Process: regulation of DNA replication (GO:0006275); Biological Process: DNA methylation (GO:0006306); Biological Process: cell proliferation (GO:0008283); Biological Process: biotin biosynthetic process (GO:0009102); Molecular Function: pyridoxal phosphate binding (GO:0030170); Biological Process: histone H3-K9 methylation (GO:0051567); Biological Process: regulation of cell cycle (GO:0051726); | Biotin metabolism (ko00780) |
| BnaC01g43920D | 9.24E-06 | -3.29171 | down | Cellular Component: nucleus (GO:0005634); Cellular Component: Golgi apparatus (GO:0005794); Biological Process: starch metabolic process (GO:0005982); Biological Process: plant-type secondary cell wall biogenesis (GO:0009834); Biological Process: glucuronoxylan biosynthetic process (GO:0010417); Molecular Function: glucuronoxylan glucuronosyltransferase activity (GO:0080116); | -- |
| BnaC02g05120D | 1.10E-19 | +∞ | up | Cellular Component: cell wall (GO:0005618); Cellular Component: mitochondrial inner membrane (GO:0005743); Cellular Component: vacuolar membrane (GO:0005774); Cellular Component: Golgi apparatus (GO:0005794); Biological Process: pentose-phosphate shunt (GO:0006098); Biological Process: transport (GO:0006810); Cellular Component: chloroplast (GO:0009507); Biological Process: response to salt stress (GO:0009651); Cellular Component: integral component of membrane (GO:0016021); | -- |
| BnaC02g06410D | 2.15E-06 | -2.98529 | down | Cellular Component: cytoplasm (GO:0005737); Cellular Component: plasmodesma (GO:0009506); Molecular Function: hydrolase activity (GO:0016787); | Glycerolipid metabolism (ko00561) |
| BnaC02g06500D | 1.11E-07 | -4.15452 | down | Molecular Function: acetolactate synthase activity (GO:0003984); Cellular Component: cytosol (GO:0005829); Biological Process: gluconeogenesis (GO:0006094); Biological Process: leucine metabolic process (GO:0006551); Biological Process: valine metabolic process (GO:0006573); Biological Process: cytoskeleton organization (GO:0007010); Biological Process: branched-chain amino acid biosynthetic process (GO:0009082); Cellular Component: chloroplast (GO:0009507); Biological Process: proteasomal protein catabolic process (GO:0010498); Molecular Function: amino acid binding (GO:0016597); | Valine, leucine and isoleucine biosynthesis (ko00290); Butanoate metabolism (ko00650); C5-Branched dibasic acid metabolism (ko00660); Pantothenate and CoA biosynthesis (ko00770); 2-Oxocarboxylic acid metabolism (ko01210); Biosynthesis of amino acids (ko01230) |
| BnaC02g06630D | 0.000144 | -3.17433 | down | -- | -- |
| BnaC02g15500D | 0.000342 | -2.12717 | down | Molecular Function: peroxidase activity (GO:0004601); Cellular Component: extracellular region (GO:0005576); Biological Process: response to oxidative stress (GO:0006979); Molecular Function: heme binding (GO:0020037); Molecular Function: metal ion binding (GO:0046872); Biological Process: oxidation-reduction process (GO:0055114); | Phenylpropanoid biosynthesis (ko00940) |
| BnaC02g18200D | 0.000594 | 2.031812 | up | -- | DNA replication (ko03030); Nucleotide excision repair (ko03420); Mismatch repair (ko03430); Homologous recombination (ko03440) |
| BnaC02g36760D | 8.40E-09 | -4.43432 | down | Molecular Function: copper ion binding (GO:0005507); Cellular Component: plasma membrane (GO:0005886); Molecular Function: electron carrier activity (GO:0009055); Biological Process: glucuronoxylan metabolic process (GO:0010413); Cellular Component: anchored component of membrane (GO:0031225); Biological Process: xylan biosynthetic process (GO:0045492); | -- |
| BnaC03g57080D | 6.96E-08 | 3.602956 | up | Cellular Component: chloroplast (GO:0009507); | -- |
| BnaC03g65980D | 1.04E-05 | -4.78954 | down | Biological Process: fatty acid biosynthetic process (GO:0006633); Cellular Component: membrane (GO:0016020); Molecular Function: transferase activity, transferring acyl groups other than amino-acyl groups (GO:0016747); | Fatty acid elongation (ko00062) |
| BnaC04g00270D | 0.000844 | -3.44033 | down | Cellular Component: mitochondrion (GO:0005739); | -- |
| BnaC04g06070D | 1.75E-08 | +∞ | up | -- | -- |
| BnaC04g18090D | 0.00057 | -3.53256 | down | Molecular Function: carboxylic ester hydrolase activity (GO:0004091); Cellular Component: extracellular region (GO:0005576); Biological Process: lipid metabolic process (GO:0006629); Biological Process: plant-type cell wall biogenesis (GO:0009832); Biological Process: glucuronoxylan metabolic process (GO:0010413); Biological Process: xylan biosynthetic process (GO:0045492); | -- |
| BnaC04g24110D | 0.000431 | -3.17482 | down | Molecular Function: polygalacturonase activity (GO:0004650); Cellular Component: extracellular region (GO:0005576); Biological Process: carbohydrate metabolic process (GO:0005975); Biological Process: cell wall modification involved in abscission (GO:0009830); Biological Process: pollen tube growth (GO:0009860); Biological Process: anther dehiscence (GO:0009901); Biological Process: fruit dehiscence (GO:0010047); | Pentose and glucuronate interconversions (ko00040); Starch and sucrose metabolism (ko00500) |
| BnaC04g39650D | 3.96E-06 | -2.39095 | down | Cellular Component: plasma membrane (GO:0005886); Cellular Component: integral component of membrane (GO:0016021); | -- |
| BnaC06g01060D | 6.38E-14 | -4.35909 | down | Molecular Function: peroxidase activity (GO:0004601); Cellular Component: extracellular region (GO:0005576); Biological Process: response to oxidative stress (GO:0006979); Molecular Function: heme binding (GO:0020037); Molecular Function: metal ion binding (GO:0046872); Biological Process: oxidation-reduction process (GO:0055114); | Phenylpropanoid biosynthesis (ko00940) |
| BnaC06g02040D | 0.000609 | 2.660511 | up | -- | -- |
| BnaC06g07780D | 5.08E-15 | -3.03356 | down | Cellular Component: extracellular region (GO:0005576); Cellular Component: mitochondrion (GO:0005739); Cellular Component: Golgi apparatus (GO:0005794); Biological Process: lignin metabolic process (GO:0009808); Biological Process: trichoblast differentiation (GO:0010054); Biological Process: glucuronoxylan metabolic process (GO:0010413); Molecular Function: glucuronoxylan 4-O-methyltransferase activity (GO:0030775); Biological Process: xylan biosynthetic process (GO:0045492); | -- |
| BnaC06g15710D | 6.65E-12 | -3.23605 | down | Cellular Component: nucleus (GO:0005634); Biological Process: response to water deprivation (GO:0009414); Biological Process: response to salt stress (GO:0009651); Biological Process: positive regulation of transcription, DNA-templated (GO:0045893); Biological Process: response to freezing (GO:0050826); | -- |
| BnaC06g16950D | 2.57E-12 | 3.968274 | up | -- | -- |
| BnaC06g30570D | 0.000412 | 3.846758 | up | Molecular Function: translation initiation factor activity (GO:0003743); Molecular Function: translation elongation factor activity (GO:0003746); Cellular Component: cytoplasm (GO:0005737); Biological Process: translational initiation (GO:0006413); Biological Process: translational frameshifting (GO:0006452); Biological Process: peptidyl-lysine modification to peptidyl-hypusine (GO:0008612); Biological Process: xylem development (GO:0010089); Molecular Function: ribosome binding (GO:0043022); Biological Process: positive regulation of translational elongation (GO:0045901); Biological Process: positive regulation of translational termination (GO:0045905); | -- |
| BnaC06g41610D | 4.58E-05 | 4.740225 | up | -- | -- |
| BnaC07g17610D | 4.51E-05 | -2.66034 | down | Cellular Component: plasma membrane (GO:0005886); Molecular Function: zinc ion binding (GO:0008270); Biological Process: plant-type secondary cell wall biogenesis (GO:0009834); Biological Process: salicylic acid mediated signaling pathway (GO:0009863); Biological Process: jasmonic acid mediated signaling pathway (GO:0009867); Biological Process: ethylene-activated signaling pathway (GO:0009873); Biological Process: glucuronoxylan metabolic process (GO:0010413); Cellular Component: integral component of membrane (GO:0016021); Molecular Function: cellulose synthase (UDP-forming) activity (GO:0016760); Biological Process: cellulose biosynthetic process (GO:0030244); Biological Process: defense response to bacterium (GO:0042742); Biological Process: xylan biosynthetic process (GO:0045492); Biological Process: defense response to fungus (GO:0050832); Biological Process: cell wall thickening (GO:0052386); | -- |
| BnaC07g24070D | 5.36E-09 | -3.73683 | down | Molecular Function: copper ion binding (GO:0005507); Cellular Component: plasma membrane (GO:0005886); Molecular Function: electron carrier activity (GO:0009055); Biological Process: glucuronoxylan metabolic process (GO:0010413); Cellular Component: anchored component of membrane (GO:0031225); Biological Process: xylan biosynthetic process (GO:0045492); | -- |
| BnaC07g36960D | 1.56E-07 | 4.294565 | up | -- | -- |
| BnaC07g50180D | 2.69E-08 | -2.9202 | down | Cellular Component: cell wall (GO:0005618); Cellular Component: plasma membrane (GO:0005886); Biological Process: response to osmotic stress (GO:0006970); Biological Process: response to water deprivation (GO:0009414); Biological Process: plant-type secondary cell wall biogenesis (GO:0009834); Biological Process: salicylic acid mediated signaling pathway (GO:0009863); Biological Process: jasmonic acid mediated signaling pathway (GO:0009867); Biological Process: ethylene-activated signaling pathway (GO:0009873); Biological Process: positive regulation of abscisic acid biosynthetic process (GO:0010116); Biological Process: glucuronoxylan metabolic process (GO:0010413); Cellular Component: integral component of membrane (GO:0016021); Molecular Function: cellulose synthase (UDP-forming) activity (GO:0016760); Biological Process: cellulose biosynthetic process (GO:0030244); Biological Process: defense response to bacterium (GO:0042742); Biological Process: xylan biosynthetic process (GO:0045492); Biological Process: defense response to fungus (GO:0050832); Biological Process: cell wall thickening (GO:0052386); | -- |
| BnaC08g01470D | 1.92E-05 | 4.522446 | up | Cellular Component: plasma membrane (GO:0005886); Biological Process: oligopeptide transport (GO:0006857); Molecular Function: kinase activity (GO:0016301); | -- |
| BnaC08g35720D | 7.53E-07 | +∞ | up | Cellular Component: vacuolar proton-transporting V-type ATPase, V0 domain (GO:0000220); Cellular Component: mitochondrion (GO:0005739); Cellular Component: Golgi apparatus (GO:0005794); Biological Process: obsolete ATP catabolic process (GO:0006200); Cellular Component: chloroplast (GO:0009507); Molecular Function: hydrogen-translocating pyrophosphatase activity (GO:0009678); Cellular Component: plant-type vacuole membrane (GO:0009705); Molecular Function: hydrogen ion transmembrane transporter activity (GO:0015078); Biological Process: ATP synthesis coupled proton transport (GO:0015986); Biological Process: ATP hydrolysis coupled proton transport (GO:0015991); Molecular Function: ATPase activity (GO:0016887); Biological Process: cellular response to nutrient levels (GO:0031669); Biological Process: sequestering of zinc ion (GO:0032119); Biological Process: vacuolar sequestering (GO:0043181); Molecular Function: nutrient reservoir activity (GO:0045735); Biological Process: vacuolar proton-transporting V-type ATPase complex assembly (GO:0070072); Biological Process: cellular response to salt stress (GO:0071472); | Oxidative phosphorylation (ko00190); Phagosome (ko04145) |
| BnaC08g36200D | 4.65E-05 | -2.68255 | down | Cellular Component: chloroplast (GO:0009507); Biological Process: photorespiration (GO:0009853); | -- |
| BnaC08g36570D | 1.44E-36 | +∞ | up | Molecular Function: actin binding (GO:0003779); Cellular Component: cell wall (GO:0005618); Cellular Component: nucleolus (GO:0005730); Cellular Component: spindle (GO:0005819); Cellular Component: cytosol (GO:0005829); Cellular Component: plasma membrane (GO:0005886); Biological Process: actin polymerization or depolymerization (GO:0008154); Cellular Component: plasmodesma (GO:0009506); Cellular Component: chloroplast (GO:0009507); Cellular Component: phragmoplast (GO:0009524); Biological Process: unidimensional cell growth (GO:0009826); Cellular Component: actin cytoskeleton (GO:0015629); Cellular Component: apoplast (GO:0048046); | -- |
| BnaC08g38300D | 4.74E-09 | -3.195 | down | Molecular Function: nucleotide binding (GO:0000166); Biological Process: mRNA splicing, via spliceosome (GO:0000398); Molecular Function: RNA binding (GO:0003723); Molecular Function: protein binding (GO:0005515); Cellular Component: nucleolus (GO:0005730); Biological Process: sugar mediated signaling pathway (GO:0010182); Cellular Component: nuclear speck (GO:0016607); | RNA transport (ko03013); mRNA surveillance pathway (ko03015) |
| BnaC08g38630D | 8.20E-10 | 3.857776 | up | Cellular Component: nucleus (GO:0005634); | -- |
| BnaC08g39040D | 2.97E-12 | +∞ | up | Molecular Function: CDP-diacylglycerol-serine O-phosphatidyltransferase activity (GO:0003882); Cellular Component: nucleus (GO:0005634); Cellular Component: mitochondrion (GO:0005739); Cellular Component: endoplasmic reticulum membrane (GO:0005789); Biological Process: phosphatidylserine biosynthetic process (GO:0006659); | Glycerophospholipid metabolism (ko00564) |
| BnaC08g39110D | 1.80E-11 | 4.651429 | up | Molecular Function: serine-type carboxypeptidase activity (GO:0004185); Cellular Component: extracellular region (GO:0005576); Cellular Component: vacuole (GO:0005773); Biological Process: proteolysis (GO:0006508); | -- |
| BnaC08g39120D | 8.67E-11 | +∞ | up | -- | -- |
| BnaC08g39130D | 2.37E-29 | +∞ | up | Molecular Function: copper ion binding (GO:0005507); Molecular Function: calmodulin binding (GO:0005516); Molecular Function: ATP binding (GO:0005524); Cellular Component: mitochondrion (GO:0005739); Cellular Component: cytosol (GO:0005829); Biological Process: gluconeogenesis (GO:0006094); Biological Process: glycolytic process (GO:0006096); Biological Process: protein folding (GO:0006457); Biological Process: tryptophan catabolic process (GO:0006569); Biological Process: response to heat (GO:0009408); Biological Process: response to cold (GO:0009409); Cellular Component: chloroplast thylakoid membrane (GO:0009535); Cellular Component: chloroplast stroma (GO:0009570); Biological Process: response to high light intensity (GO:0009644); Biological Process: response to salt stress (GO:0009651); Biological Process: chloroplast organization (GO:0009658); Biological Process: indoleacetic acid biosynthetic process (GO:0009684); Cellular Component: chloroplast envelope (GO:0009941); Biological Process: isopentenyl diphosphate biosynthetic process, methylerythritol 4-phosphate pathway (GO:0019288); Biological Process: cysteine biosynthetic process (GO:0019344); Biological Process: response to endoplasmic reticulum stress (GO:0034976); Biological Process: response to hydrogen peroxide (GO:0042542); Biological Process: response to cadmium ion (GO:0046686); Cellular Component: apoplast (GO:0048046); Biological Process: plant ovule development (GO:0048481); Molecular Function: chaperone binding (GO:0051087); Biological Process: positive regulation of superoxide dismutase activity (GO:1901671); | -- |
| BnaC08g39240D | 0.000126 | -2.38157 | down | Biological Process: RNA methylation (GO:0001510); Molecular Function: nucleocytoplasmic transporter activity (GO:0005487); Molecular Function: protein binding (GO:0005515); Cellular Component: nuclear pore (GO:0005643); Cellular Component: nucleolus (GO:0005730); Cellular Component: plasma membrane (GO:0005886); Biological Process: nucleocytoplasmic transport (GO:0006913); Cellular Component: plasmodesma (GO:0009506); Cellular Component: chloroplast (GO:0009507); Molecular Function: structural constituent of nuclear pore (GO:0017056); | RNA transport (ko03013) |
| BnaC08g39360D | 1.14E-06 | +∞ | up | Molecular Function: hydrolase activity, hydrolyzing O-glycosyl compounds (GO:0004553); Cellular Component: cell wall (GO:0005618); Biological Process: cellular glucan metabolic process (GO:0006073); Biological Process: phloem or xylem histogenesis (GO:0010087); Biological Process: fruit development (GO:0010154); Molecular Function: xyloglucan:xyloglucosyl transferase activity (GO:0016762); Cellular Component: apoplast (GO:0048046); Biological Process: stamen filament development (GO:0080086); | -- |
| BnaC08g39400D | 0.000144 | 3.496442 | up | Molecular Function: DNA binding (GO:0003677); Molecular Function: transcription factor activity, sequence-specific DNA binding (GO:0003700); Cellular Component: nucleus (GO:0005634); Biological Process: regulation of transcription, DNA-templated (GO:0006355); Biological Process: response to ethylene (GO:0009723); Biological Process: regulation of developmental process (GO:0050793); | -- |
| BnaC08g39990D | 1.12E-05 | 4.097042 | up | Biological Process: MAPK cascade (GO:0000165); Molecular Function: protein serine/threonine kinase activity (GO:0004674); Molecular Function: protein serine/threonine/tyrosine kinase activity (GO:0004712); Molecular Function: ATP binding (GO:0005524); Cellular Component: nucleus (GO:0005634); Cellular Component: cytosol (GO:0005829); Biological Process: protein phosphorylation (GO:0006468); Biological Process: protein targeting to membrane (GO:0006612); Biological Process: response to cold (GO:0009409); Biological Process: response to water deprivation (GO:0009414); Biological Process: response to ethylene (GO:0009723); Biological Process: auxin-activated signaling pathway (GO:0009734); Biological Process: abscisic acid-activated signaling pathway (GO:0009738); Biological Process: brassinosteroid mediated signaling pathway (GO:0009742); Biological Process: systemic acquired resistance, salicylic acid mediated signaling pathway (GO:0009862); Biological Process: jasmonic acid mediated signaling pathway (GO:0009867); Biological Process: regulation of signal transduction (GO:0009966); Biological Process: leaf vascular tissue pattern formation (GO:0010305); Biological Process: regulation of plant-type hypersensitive response (GO:0010363); Biological Process: endoplasmic reticulum unfolded protein response (GO:0030968); Biological Process: negative regulation of defense response (GO:0031348); Biological Process: hyperosmotic salinity response (GO:0042538); Biological Process: negative regulation of programmed cell death (GO:0043069); Biological Process: defense response to fungus (GO:0050832); | -- |
| BnaC08g40400D | 4.03E-06 | 4.446284 | up | Molecular Function: DNA binding (GO:0003677); Cellular Component: nucleus (GO:0005634); Cellular Component: Golgi apparatus (GO:0005794); Cellular Component: cytosol (GO:0005829); Biological Process: glucose catabolic process (GO:0006007); Molecular Function: GTPase activator activity (GO:0008060); Molecular Function: zinc ion binding (GO:0008270); Biological Process: cellulose biosynthetic process (GO:0030244); Biological Process: regulation of GTPase activity (GO:0032312); Biological Process: Golgi vesicle transport (GO:0048193); | Endocytosis (ko04144) |
| BnaC08g40410D | 2.05E-22 | +∞ | up | Molecular Function: GTPase activator activity (GO:0005098); Cellular Component: nuclear envelope (GO:0005635); Cellular Component: vacuolar membrane (GO:0005774); Cellular Component: endoplasmic reticulum (GO:0005783); Biological Process: nucleocytoplasmic transport (GO:0006913); Biological Process: toxin catabolic process (GO:0009407); Cellular Component: chloroplast (GO:0009507); Biological Process: photomorphogenesis (GO:0009640); Biological Process: response to salt stress (GO:0009651); Biological Process: protein deneddylation (GO:0010388); Biological Process: lateral root development (GO:0048527); | RNA transport (ko03013) |
| BnaC08g40810D | 4.86E-09 | 2.406417 | up | Molecular Function: protein serine/threonine kinase activity (GO:0004674); Biological Process: protein autophosphorylation (GO:0046777); | -- |
| BnaC08g41180D | 0.000886 | -2.5698 | down | Molecular Function: DNA binding (GO:0003677); Cellular Component: nucleus (GO:0005634); Molecular Function: zinc ion binding (GO:0008270); | -- |
| BnaC08g41210D | 1.39E-05 | 2.677377 | up | -- | -- |
| BnaC08g41290D | 1.40E-05 | -3.47156 | down | Molecular Function: nucleic acid binding (GO:0003676); Cellular Component: nucleus (GO:0005634); Cellular Component: chloroplast (GO:0009507); | -- |
| BnaC08g41720D | 2.67E-05 | -2.69705 | down | Molecular Function: aspartic-type endopeptidase activity (GO:0004190); Cellular Component: extracellular region (GO:0005576); Cellular Component: vacuole (GO:0005773); Cellular Component: cytosol (GO:0005829); Biological Process: glycolytic process (GO:0006096); Biological Process: proteolysis (GO:0006508); Biological Process: protein targeting to vacuole (GO:0006623); Biological Process: lipid metabolic process (GO:0006629); Biological Process: water transport (GO:0006833); Biological Process: hyperosmotic response (GO:0006972); Biological Process: Golgi organization (GO:0007030); Biological Process: response to temperature stimulus (GO:0009266); Cellular Component: plasmodesma (GO:0009506); Biological Process: response to salt stress (GO:0009651); Biological Process: response to cadmium ion (GO:0046686); Biological Process: animal organ development (GO:0048513); | -- |
| BnaC08g42280D | 1.20E-07 | -3.798 | down | Biological Process: telomere maintenance (GO:0000723); Biological Process: double-strand break repair via homologous recombination (GO:0000724); Molecular Function: nucleic acid binding (GO:0003676); Molecular Function: ATP binding (GO:0005524); Cellular Component: nucleus (GO:0005634); Biological Process: DNA replication (GO:0006260); Cellular Component: plasmodesma (GO:0009506); Biological Process: vegetative to reproductive phase transition of meristem (GO:0010228); Molecular Function: ATP-dependent 3'-5' DNA helicase activity (GO:0043140); Biological Process: cellular response to cold (GO:0070417); Biological Process: cellular response to abscisic acid stimulus (GO:0071215); | Homologous recombination (ko03440) |
| BnaC08g49610D | 5.83E-07 | -2.84759 | down | Molecular Function: protein binding (GO:0005515); Molecular Function: ATP binding (GO:0005524); Cellular Component: mitochondrion (GO:0005739); Biological Process: starch catabolic process (GO:0005983); Biological Process: circadian rhythm (GO:0007623); Cellular Component: chloroplast stroma (GO:0009570); Biological Process: response to symbiotic fungus (GO:0009610); Biological Process: cold acclimation (GO:0009631); Cellular Component: chloroplast envelope (GO:0009941); Biological Process: phosphorylation (GO:0016310); Biological Process: starch biosynthetic process (GO:0019252); Molecular Function: alpha-glucan, water dikinase activity (GO:0050521); | -- |
| BnaC09g05400D | 2.10E-14 | +∞ | up | Cellular Component: nucleus (GO:0005634); | -- |
| BnaC09g05590D | 4.85E-05 | 2.881326 | up | Molecular Function: pectinesterase activity (GO:0030599); Biological Process: negative regulation of catalytic activity (GO:0043086); Molecular Function: pectinesterase inhibitor activity (GO:0046910); | -- |
| BnaC09g05960D | 1.37E-07 | 5.221473 | up | Molecular Function: DNA binding (GO:0003677); Cellular Component: nucleus (GO:0005634); | -- |
| BnaC09g06220D | 6.77E-20 | 4.951949 | up | Cellular Component: plasma membrane (GO:0005886); Cellular Component: chloroplast (GO:0009507); | -- |
| BnaC09g06260D | 1.07E-31 | +∞ | up | Cellular Component: nucleus (GO:0005634); | -- |
| BnaC09g31020D | 1.12E-05 | -3.04999 | down | Cellular Component: Golgi apparatus (GO:0005794); Biological Process: plant-type cell wall biogenesis (GO:0009832); Biological Process: xylem development (GO:0010089); Biological Process: glucuronoxylan biosynthetic process (GO:0010417); Molecular Function: polygalacturonate 4-alpha-galacturonosyltransferase activity (GO:0047262); Biological Process: cell wall organization (GO:0071555); | Starch and sucrose metabolism (ko00500); Amino sugar and nucleotide sugar metabolism (ko00520) |
| BnaC09g37510D | 3.03E-12 | 4.355824 | up | Molecular Function: hydrolase activity, hydrolyzing O-glycosyl compounds (GO:0004553); Biological Process: response to oxidative stress (GO:0006979); Biological Process: metabolic process (GO:0008152); Biological Process: response to cold (GO:0009409); Cellular Component: plasmodesma (GO:0009506); Cellular Component: chloroplast (GO:0009507); Biological Process: response to sucrose (GO:0009744); Biological Process: response to fructose (GO:0009750); Biological Process: response to karrikin (GO:0080167); | Galactose metabolism (ko00052) |
| BnaC09g53990D | 3.02E-23 | +∞ | up | Biological Process: sulfur amino acid metabolic process (GO:0000096); Biological Process: MAPK cascade (GO:0000165); Molecular Function: iron ion binding (GO:0005506); Cellular Component: nucleus (GO:0005634); Biological Process: pentose-phosphate shunt (GO:0006098); Biological Process: regulation of translation (GO:0006417); Biological Process: glycine catabolic process (GO:0006546); Biological Process: protein targeting to membrane (GO:0006612); Biological Process: unsaturated fatty acid biosynthetic process (GO:0006636); Biological Process: phosphatidylglycerol biosynthetic process (GO:0006655); Biological Process: vitamin metabolic process (GO:0006766); Biological Process: cellular amino acid biosynthetic process (GO:0008652); Biological Process: aromatic amino acid family metabolic process (GO:0009072); Biological Process: lipoate metabolic process (GO:0009106); Biological Process: coenzyme biosynthetic process (GO:0009108); Biological Process: response to cold (GO:0009409); Cellular Component: chloroplast stroma (GO:0009570); Biological Process: detection of biotic stimulus (GO:0009595); Biological Process: response to blue light (GO:0009637); Biological Process: response to high light intensity (GO:0009644); Biological Process: plastid organization (GO:0009657); Biological Process: jasmonic acid biosynthetic process (GO:0009695); Biological Process: salicylic acid biosynthetic process (GO:0009697); Biological Process: response to sucrose (GO:0009744); Biological Process: systemic acquired resistance, salicylic acid mediated signaling pathway (GO:0009862); Biological Process: jasmonic acid mediated signaling pathway (GO:0009867); Cellular Component: chloroplast envelope (GO:0009941); Biological Process: response to red light (GO:0010114); Biological Process: regulation of proton transport (GO:0010155); Biological Process: response to chitin (GO:0010200); Biological Process: response to far red light (GO:0010218); Biological Process: PSII associated light-harvesting complex II catabolic process (GO:0010304); Biological Process: regulation of hydrogen peroxide metabolic process (GO:0010310); Biological Process: regulation of plant-type hypersensitive response (GO:0010363); Biological Process: chlorophyll biosynthetic process (GO:0015995); Biological Process: carotenoid biosynthetic process (GO:0016117); Biological Process: regulation of lipid metabolic process (GO:0019216); Biological Process: starch biosynthetic process (GO:0019252); Biological Process: isopentenyl diphosphate biosynthetic process, methylerythritol 4-phosphate pathway (GO:0019288); Biological Process: photosynthesis, light reaction (GO:0019684); Biological Process: glucosinolate metabolic process (GO:0019760); Biological Process: negative regulation of defense response (GO:0031348); Biological Process: oxylipin biosynthetic process (GO:0031408); Biological Process: defense response to bacterium (GO:0042742); Biological Process: regulation of multi-organism process (GO:0043900); Biological Process: sulfur compound biosynthetic process (GO:0044272); Biological Process: positive regulation of transcription, DNA-templated (GO:0045893); Molecular Function: 4-hydroxy-3-methylbut-2-en-1-yl diphosphate synthase activity (GO:0046429); Biological Process: defense response to fungus (GO:0050832); Molecular Function: 4 iron, 4 sulfur cluster binding (GO:0051539); | Terpenoid backbone biosynthesis (ko00900) |
| BnaCnng08170D | 0.000515 | -4.83278 | down | Cellular Component: extracellular region (GO:0005576); Biological Process: response to other organism (GO:0051707); | -- |
| BnaCnng17490D | 8.87E-15 | 3.632534 | up | -- | -- |
| BnaCnng24040D | 5.53E-05 | 4.922701 | up | Molecular Function: protein binding (GO:0005515); Cellular Component: cytosol (GO:0005829); Biological Process: glycolytic process (GO:0006096); Biological Process: tricarboxylic acid cycle (GO:0006099); Biological Process: iron ion transport (GO:0006826); Biological Process: water transport (GO:0006833); Biological Process: hyperosmotic response (GO:0006972); Biological Process: Golgi organization (GO:0007030); Molecular Function: phosphoenolpyruvate carboxylase activity (GO:0008964); Biological Process: response to temperature stimulus (GO:0009266); Biological Process: response to salt stress (GO:0009651); Biological Process: response to sucrose (GO:0009744); Biological Process: response to glucose (GO:0009749); Biological Process: response to fructose (GO:0009750); Biological Process: cellular response to iron ion starvation (GO:0010106); Biological Process: response to nitrate (GO:0010167); Biological Process: nitrate transport (GO:0015706); Biological Process: carbon fixation (GO:0015977); Biological Process: cellular response to phosphate starvation (GO:0016036); Biological Process: response to cadmium ion (GO:0046686); Cellular Component: apoplast (GO:0048046); Biological Process: protein tetramerization (GO:0051262); | Pyruvate metabolism (ko00620); Carbon fixation in photosynthetic organisms (ko00710); Carbon metabolism (ko01200) |
| BnaCnng36050D | 0.000739 | 3.7552 | up | -- | -- |
| Brassica_napus_newGene_10149 | 4.02E-05 | 2.757749 | up | -- | -- |
| Brassica_napus_newGene_10370 | 0.000131 | -3.14855 | down | -- | -- |
| Brassica_napus_newGene_10523 | 0.000628 | 2.260291 | up | -- | -- |
| Brassica_napus_newGene_11035 | 2.99E-06 | 7.464209 | up | Molecular Function: hydrolase activity, acting on ester bonds (GO:0016788); | -- |
| Brassica_napus_newGene_12095 | 4.96E-17 | +∞ | up | Cellular Component: cell wall (GO:0005618); Cellular Component: vacuole (GO:0005773); Cellular Component: endoplasmic reticulum (GO:0005783); Cellular Component: plasma membrane (GO:0005886); Cellular Component: plasmodesma (GO:0009506); Cellular Component: integral component of membrane (GO:0016021); Molecular Function: transmembrane transporter activity (GO:0022857); Biological Process: transmembrane transport (GO:0055085); | -- |
| Brassica_napus_newGene_12198 | 8.87E-11 | 2.537667 | up | -- | -- |
| Brassica_napus_newGene_1404 | 1.30E-09 | -6.56313 | down | Molecular Function: transmembrane receptor protein serine/threonine kinase activity (GO:0004675); Molecular Function: ATP binding (GO:0005524); Cellular Component: integral component of plasma membrane (GO:0005887); Biological Process: cell surface receptor signaling pathway (GO:0007166); Biological Process: response to wounding (GO:0009611); Biological Process: response to fungus (GO:0009620); Biological Process: protein autophosphorylation (GO:0046777); | -- |
| Brassica_napus_newGene_2419 | 1.59E-06 | -4.08059 | down | -- | -- |
| Brassica_napus_newGene_2420 | 7.21E-07 | -4.14464 | down | -- | -- |
| Brassica_napus_newGene_3285 | 4.87E-09 | -3.00162 | down | -- | -- |
| Brassica_napus_newGene_3308 | 3.34E-23 | +∞ | up | Molecular Function: ATP binding (GO:0005524); Cellular Component: cell wall (GO:0005618); Cellular Component: mitochondrion (GO:0005739); Cellular Component: chloroplast stroma (GO:0009570); | Protein processing in endoplasmic reticulum (ko04141); Plant-pathogen interaction (ko04626) |
| Brassica_napus_newGene_3329 | 2.35E-44 | 4.111738 | up | -- | -- |
| Brassica_napus_newGene_3344 | 0.000416 | -2.95656 | down | -- | -- |
| Brassica_napus_newGene_3472 | 1.68E-05 | -2.73816 | down | Cellular Component: plasma membrane (GO:0005886); Cellular Component: integral component of endoplasmic reticulum membrane (GO:0030176); | Protein export (ko03060); Protein processing in endoplasmic reticulum (ko04141) |
| Brassica_napus_newGene_6758 | 6.97E-38 | -4.17218 | down | -- | -- |
| Brassica_napus_newGene_695 | 1.95E-05 | 2.309219 | up | Cellular Component: extracellular region (GO:0005576); Cellular Component: plant-type cell wall (GO:0009505); Cellular Component: integral component of membrane (GO:0016021); Molecular Function: pectinesterase activity (GO:0030599); Biological Process: cell wall modification (GO:0042545); Biological Process: negative regulation of catalytic activity (GO:0043086); Molecular Function: aspartyl esterase activity (GO:0045330); Biological Process: pectin catabolic process (GO:0045490); Molecular Function: pectinesterase inhibitor activity (GO:0046910); Cellular Component: pollen tube (GO:0090406); | -- |
| Brassica_napus_newGene_7257 | 3.74E-35 | 4.031021 | up | Molecular Function: nucleic acid binding (GO:0003676); Molecular Function: zinc ion binding (GO:0008270); | -- |
| Brassica_napus_newGene_8007 | 1.24E-13 | +∞ | up | -- | -- |
| Brassica_napus_newGene_81 | 3.64E-08 | 2.041156 | up | -- | -- |
| Brassica_napus_newGene_8461 | 0.000677 | 2.014625 | up | Molecular Function: structural constituent of ribosome (GO:0003735); Biological Process: translation (GO:0006412); Cellular Component: chloroplast (GO:0009507); Cellular Component: small ribosomal subunit (GO:0015935); Molecular Function: rRNA binding (GO:0019843); | Ribosome (ko03010) |
| Brassica_napus_newGene_87 | 0.000476 | 3.108932 | up | Cellular Component: integral component of membrane (GO:0016021); | -- |
