# Supplementary material for: Investigation of Thermomorphogenesis-Related Genes for a Multi-Silique Trait in Brassica napus by Comparative Transcriptome Analysis
Source: Front Genet. 2021 Jul 23;12:678804. doi: 10.3389/fgene.2021.678804 (PMC8343136; doi:10.3389/fgene.2021.678804)
Supplement: Supplementary Table 2 — Line-specific expressed genes from Xindu. [file Table_2.DOCX]

**Supplementary Table 2|** Line-specific expressed genes from Xindu.

| Gene ID | FDR | log_2_FC | GO annotation | KEGG pathway annotation |
| --- | --- | --- | --- | --- |
| BnaCnng75420D | 8.59E-14 | +∞ | Cellular Component: vacuolar proton-transporting V-type ATPase, V0 domain (GO:0000220); Cellular Component: mitochondrion (GO:0005739); Cellular Component: Golgi apparatus (GO:0005794); Biological Process: ATP catabolic process (GO:0006200); Cellular Component: chloroplast (GO:0009507); Molecular Function: hydrogen-translocating pyrophosphatase activity (GO:0009678); Cellular Component: plant-type vacuole membrane (GO:0009705); Molecular Function: hydrogen ion transmembrane transporter activity (GO:0015078); Biological Process: ATP synthesis coupled proton transport (GO:0015986); Biological Process: ATP hydrolysis coupled proton transport (GO:0015991); Molecular Function: ATPase activity (GO:0016887); Biological Process: cellular response to nutrient levels (GO:0031669); Biological Process: sequestering of zinc ion (GO:0032119); Biological Process: vacuolar sequestering (GO:0043181); Molecular Function: nutrient reservoir activity (GO:0045735); Biological Process: vacuolar proton-transporting V-type ATPase complex assembly (GO:0070072); Biological Process: cellular response to salt stress (GO:0071472); | Oxidative phosphorylation (ko00190); Phagosome (ko04145) |
| BnaC08g40410D | 1.08E-13 | +∞ | Molecular Function: Ran GTPase activator activity (GO:0005098); Cellular Component: nuclear envelope (GO:0005635); Cellular Component: vacuolar membrane (GO:0005774); Cellular Component: endoplasmic reticulum (GO:0005783); Biological Process: nucleocytoplasmic transport (GO:0006913); Biological Process: toxin catabolic process (GO:0009407); Cellular Component: chloroplast (GO:0009507); Biological Process: photomorphogenesis (GO:0009640); Biological Process: response to salt stress (GO:0009651); Biological Process: cullin deneddylation (GO:0010388); Biological Process: lateral root development (GO:0048527); | RNA transport (ko03013) |
| BnaC08g39020D | 5.05E-25 | +∞ | Cellular Component: cytosol (GO:0005829); Cellular Component: plasmodesma (GO:0009506); | Endocytosis (ko04144) |
| Cole_newGene_2073 | 1.79E-77 | +∞ | Cellular Component: intracellular membrane-bounded organelle (GO:0043231); Cellular Component: cytoplasmic part (GO:0044444); | Protein processing in endoplasmic reticulum (ko04141); Plant-pathogen interaction (ko04626) |
| BnaC04g30490D | 4.13E-16 | +∞ | -- | -- |
| BnaC08g37340D | 1.27E-09 | +∞ | Cellular Component: plasma membrane (GO:0005886); Biological Process: proteolysis (GO:0006508); Biological Process: lipid transport (GO:0006869); Molecular Function: peptidase activity (GO:0008233); Molecular Function: lipid binding (GO:0008289); Cellular Component: anchored component of membrane (GO:0031225); | -- |
| BnaC04g30180D | 1.39E-05 | +∞ | -- | -- |
| BnaA04g06410D | 2.11E-15 | +∞ | Biological Process: heme biosynthetic process (GO:0006783); Biological Process: response to oxidative stress (GO:0006979); Molecular Function: glutamyl-tRNA reductase activity (GO:0008883); Cellular Component: chloroplast (GO:0009507); Biological Process: response to wounding (GO:0009611); Biological Process: response to chitin (GO:0010200); Biological Process: defense response by callose deposition (GO:0052542); | Porphyrin and chlorophyll metabolism (ko00860) |
| Cole_newGene_2682 | 1.63E-10 | +∞ | -- | -- |
| BnaC08g39120D | 1.30E-10 | +∞ | -- | -- |
| BnaC08g49500D | 6.27E-15 | +∞ | Cellular Component: chloroplast (GO:0009507); Biological Process: systemic acquired resistance (GO:0009627); Biological Process: regulation of defense response (GO:0031347); | -- |
| BnaC09g05590D | 6.70E-08 | +∞ | Molecular Function: pectinesterase activity (GO:0030599); Biological Process: negative regulation of catalytic activity (GO:0043086); Molecular Function: pectinesterase inhibitor activity (GO:0046910); | -- |
| Cole_newGene_6687 | 4.72E-06 | +∞ | Cellular Component: cell wall (GO:0005618); Cellular Component: vacuole (GO:0005773); Cellular Component: endoplasmic reticulum (GO:0005783); Cellular Component: plasma membrane (GO:0005886); Cellular Component: plasmodesma (GO:0009506); Molecular Function: transmembrane transporter activity (GO:0022857); Biological Process: transmembrane transport (GO:0055085); | -- |
| BnaC09g05960D | 1.38E-09 | +∞ | Molecular Function: DNA binding (GO:0003677); Cellular Component: nucleus (GO:0005634); | -- |
| BnaC08g35720D | 3.22E-25 | +∞ | Cellular Component: vacuolar proton-transporting V-type ATPase, V0 domain (GO:0000220); Cellular Component: mitochondrion (GO:0005739); Cellular Component: Golgi apparatus (GO:0005794); Biological Process: ATP catabolic process (GO:0006200); Cellular Component: chloroplast (GO:0009507); Molecular Function: hydrogen-translocating pyrophosphatase activity (GO:0009678); Cellular Component: plant-type vacuole membrane (GO:0009705); Molecular Function: hydrogen ion transmembrane transporter activity (GO:0015078); Biological Process: ATP synthesis coupled proton transport (GO:0015986); Biological Process: ATP hydrolysis coupled proton transport (GO:0015991); Molecular Function: ATPase activity (GO:0016887); Biological Process: cellular response to nutrient levels (GO:0031669); Biological Process: sequestering of zinc ion (GO:0032119); Biological Process: vacuolar sequestering (GO:0043181); Molecular Function: nutrient reservoir activity (GO:0045735); Biological Process: vacuolar proton-transporting V-type ATPase complex assembly (GO:0070072); Biological Process: cellular response to salt stress (GO:0071472); | Oxidative phosphorylation (ko00190); Phagosome (ko04145) |
| BnaC08g39130D | 5.02E-80 | +∞ | Molecular Function: copper ion binding (GO:0005507); Molecular Function: calmodulin binding (GO:0005516); Molecular Function: ATP binding (GO:0005524); Cellular Component: mitochondrion (GO:0005739); Cellular Component: cytosol (GO:0005829); Biological Process: gluconeogenesis (GO:0006094); Biological Process: glycolytic process (GO:0006096); Biological Process: protein folding (GO:0006457); Biological Process: tryptophan catabolic process (GO:0006569); Biological Process: response to heat (GO:0009408); Biological Process: response to cold (GO:0009409); Cellular Component: chloroplast thylakoid membrane (GO:0009535); Cellular Component: chloroplast stroma (GO:0009570); Biological Process: response to high light intensity (GO:0009644); Biological Process: response to salt stress (GO:0009651); Biological Process: chloroplast organization (GO:0009658); Biological Process: indoleacetic acid biosynthetic process (GO:0009684); Cellular Component: chloroplast envelope (GO:0009941); Biological Process: isopentenyl diphosphate biosynthetic process, methylerythritol 4-phosphate pathway (GO:0019288); Biological Process: cysteine biosynthetic process (GO:0019344); Biological Process: response to endoplasmic reticulum stress (GO:0034976); Biological Process: response to hydrogen peroxide (GO:0042542); Biological Process: response to cadmium ion (GO:0046686); Cellular Component: apoplast (GO:0048046); Biological Process: ovule development (GO:0048481); Molecular Function: chaperone binding (GO:0051087); Biological Process: positive regulation of superoxide dismutase activity (GO:1901671); | -- |
| BnaC09g06110D | 1.65E-09 | +∞ | Cellular Component: extracellular region (GO:0005576); Cellular Component: endoplasmic reticulum (GO:0005783); Cellular Component: chloroplast (GO:0009507); Biological Process: unidimensional cell growth (GO:0009826); Molecular Function: glucosidase activity (GO:0015926); Biological Process: cellulose biosynthetic process (GO:0030244); Molecular Function: carbohydrate binding (GO:0030246); Biological Process: defense response to bacterium (GO:0042742); Biological Process: response to cadmium ion (GO:0046686); Biological Process: Golgi vesicle transport (GO:0048193); | N-Glycan biosynthesis (ko00510); Protein processing in endoplasmic reticulum (ko04141) |
| BnaC04g29730D | 4.12E-16 | +∞ | Cellular Component: cytosol (GO:0005829); Biological Process: response to wounding (GO:0009611); Biological Process: response to salicylic acid (GO:0009751); Molecular Function: 12-oxophytodienoate reductase activity (GO:0016629); Biological Process: oxylipin metabolic process (GO:0031407); Biological Process: response to cadmium ion (GO:0046686); | alpha-Linolenic acid metabolism (ko00592) |
| BnaC08g40320D | 3.98E-17 | +∞ | Molecular Function: chromatin binding (GO:0003682); Molecular Function: sequence-specific DNA binding transcription factor activity (GO:0003700); Cellular Component: nucleus (GO:0005634); Biological Process: regulation of transcription, DNA-templated (GO:0006355); Biological Process: membrane fusion (GO:0006944); Molecular Function: identical protein binding (GO:0042802); Molecular Function: sequence-specific DNA binding (GO:0043565); Biological Process: Golgi vesicle transport (GO:0048193); | -- |
| BnaA09g06740D | 1.47E-10 | +∞ | Cellular Component: nucleus (GO:0005634); | -- |
| BnaC01g43270D | 1.86E-07 | +∞ | Molecular Function: magnesium ion binding (GO:0000287); Molecular Function: adenosylmethionine-8-amino-7-oxononanoate transaminase activity (GO:0004015); Molecular Function: dethiobiotin synthase activity (GO:0004141); Molecular Function: ATP binding (GO:0005524); Cellular Component: mitochondrion (GO:0005739); Biological Process: DNA replication initiation (GO:0006270); Biological Process: regulation of DNA replication (GO:0006275); Biological Process: DNA methylation (GO:0006306); Biological Process: cell proliferation (GO:0008283); Biological Process: biotin biosynthetic process (GO:0009102); Molecular Function: pyridoxal phosphate binding (GO:0030170); Biological Process: histone H3-K9 methylation (GO:0051567); Biological Process: regulation of cell cycle (GO:0051726); | Biotin metabolism (ko00780) |
| BnaC08g42280D | 1.47E-13 | -∞ | Biological Process: telomere maintenance (GO:0000723); Biological Process: double-strand break repair via homologous recombination (GO:0000724); Molecular Function: nucleic acid binding (GO:0003676); Molecular Function: ATP binding (GO:0005524); Cellular Component: nucleus (GO:0005634); Biological Process: DNA replication (GO:0006260); Cellular Component: plasmodesma (GO:0009506); Biological Process: vegetative to reproductive phase transition of meristem (GO:0010228); Molecular Function: ATP-dependent 3'-5' DNA helicase activity (GO:0043140); Biological Process: cellular response to cold (GO:0070417); Biological Process: cellular response to abscisic acid stimulus (GO:0071215); | Homologous recombination (ko03440) |
| BnaC08g41780D | 2.03E-10 | -∞ | Biological Process: sulfur amino acid metabolic process (GO:0000096); Molecular Function: serine-tRNA ligase activity (GO:0004828); Molecular Function: ATP binding (GO:0005524); Cellular Component: mitochondrion (GO:0005739); Biological Process: rRNA processing (GO:0006364); Biological Process: seryl-tRNA aminoacylation (GO:0006434); Biological Process: mitochondrion organization (GO:0007005); Biological Process: cellular amino acid biosynthetic process (GO:0008652); Biological Process: serine family amino acid metabolic process (GO:0009069); Cellular Component: chloroplast (GO:0009507); Biological Process: embryo development ending in seed dormancy (GO:0009793); Biological Process: chloroplast relocation (GO:0009902); Biological Process: leaf morphogenesis (GO:0009965); Biological Process: thylakoid membrane organization (GO:0010027); Biological Process: photosystem II assembly (GO:0010207); Biological Process: vegetative to reproductive phase transition of meristem (GO:0010228); Biological Process: iron-sulfur cluster assembly (GO:0016226); Biological Process: cell differentiation (GO:0030154); Biological Process: regulation of protein dephosphorylation (GO:0035304); Biological Process: cell wall modification (GO:0042545); Biological Process: transcription from plastid promoter (GO:0042793); Biological Process: positive regulation of transcription, DNA-templated (GO:0045893); Biological Process: ovule development (GO:0048481); | Aminoacyl-tRNA biosynthesis (ko00970) |
| BnaC02g06570D | 4.64E-14 | -∞ | Biological Process: maltose metabolic process (GO:0000023); Molecular Function: enzyme inhibitor activity (GO:0004857); Cellular Component: cytosol (GO:0005829); Biological Process: pentose-phosphate shunt (GO:0006098); Biological Process: regulation of carbohydrate metabolic process (GO:0006109); Biological Process: glycerol ether metabolic process (GO:0006662); Molecular Function: enzyme activator activity (GO:0008047); Molecular Function: electron carrier activity (GO:0009055); Cellular Component: chloroplast thylakoid (GO:0009534); Cellular Component: chloroplast stroma (GO:0009570); Molecular Function: protein disulfide oxidoreductase activity (GO:0015035); Biological Process: starch biosynthetic process (GO:0019252); Biological Process: isopentenyl diphosphate biosynthetic process, methylerythritol 4-phosphate pathway (GO:0019288); Biological Process: glucosinolate metabolic process (GO:0019760); Biological Process: electron transport chain (GO:0022900); Biological Process: positive regulation of catalytic activity (GO:0043085); Biological Process: negative regulation of catalytic activity (GO:0043086); Biological Process: cell redox homeostasis (GO:0045454); | -- |
| Cole_newGene_1983 | 7.64E-16 | -∞ | Cellular Component: mitochondrion (GO:0005739); Cellular Component: chloroplast stroma (GO:0009570); Biological Process: mRNA modification (GO:0016556); Molecular Function: carbon-nitrogen ligase activity, with glutamine as amido-N-donor (GO:0016884); | Aminoacyl-tRNA biosynthesis (ko00970) |
| BnaC02g06360D | 7.18E-08 | -∞ | Molecular Function: 3-oxo-5-alpha-steroid 4-dehydrogenase activity (GO:0003865); Biological Process: lipid metabolic process (GO:0006629); Cellular Component: chloroplast envelope (GO:0009941); Cellular Component: integral component of membrane (GO:0016021); | -- |
| Cole_newGene_4151 | 8.92E-179 | -∞ | -- | -- |
| Cole_newGene_2071 | 8.38E-27 | -∞ | Molecular Function: sequence-specific DNA binding transcription factor activity (GO:0003700); Cellular Component: nucleus (GO:0005634); Biological Process: regulation of transcription, DNA-templated (GO:0006355); Molecular Function: protein dimerization activity (GO:0046983); | -- |
| BnaC08g38300D | 6.73E-24 | -∞ | Molecular Function: nucleotide binding (GO:0000166); Biological Process: mRNA splicing, via spliceosome (GO:0000398); Molecular Function: RNA binding (GO:0003723); Molecular Function: protein binding (GO:0005515); Cellular Component: nucleolus (GO:0005730); Biological Process: sugar mediated signaling pathway (GO:0010182); Cellular Component: nuclear speck (GO:0016607); | RNA transport (ko03013); mRNA surveillance pathway (ko03015) |
| BnaA09g47900D | 2.97E-12 | -∞ | Molecular Function: zinc ion binding (GO:0008270); | -- |
| BnaC08g41390D | 1.90E-08 | -∞ | Cellular Component: plant-type vacuole (GO:0000325); Molecular Function: sucrose alpha-glucosidase activity (GO:0004575); Biological Process: carbohydrate metabolic process (GO:0005975); Biological Process: polyamine catabolic process (GO:0006598); Biological Process: calcium ion transport (GO:0006816); Biological Process: iron ion transport (GO:0006826); Biological Process: Golgi organization (GO:0007030); Cellular Component: plant-type cell wall (GO:0009505); Biological Process: response to wounding (GO:0009611); Biological Process: response to bacterium (GO:0009617); Biological Process: response to salt stress (GO:0009651); Biological Process: coumarin biosynthetic process (GO:0009805); Biological Process: cellular response to iron ion starvation (GO:0010106); Biological Process: response to nitrate (GO:0010167); Biological Process: nitrate transport (GO:0015706); Biological Process: brassinosteroid biosynthetic process (GO:0016132); Biological Process: cellular modified amino acid biosynthetic process (GO:0042398); Biological Process: cellular response to gibberellin stimulus (GO:0071370); Biological Process: primary root development (GO:0080022); | Galactose metabolism (ko00052); Starch and sucrose metabolism (ko00500) |
| BnaC02g06410D | 1.26E-06 | -∞ | Cellular Component: cytoplasm (GO:0005737); Cellular Component: plasmodesma (GO:0009506); Molecular Function: hydrolase activity (GO:0016787); | -- |
| BnaC03g24650D | 1.59E-10 | -∞ | Biological Process: cell morphogenesis (GO:0000902); Cellular Component: nucleus (GO:0005634); Cellular Component: Golgi apparatus (GO:0005794); Cellular Component: cytosol (GO:0005829); Cellular Component: plasma membrane (GO:0005886); Biological Process: gluconeogenesis (GO:0006094); Biological Process: glycolytic process (GO:0006096); Biological Process: response to desiccation (GO:0009269); Cellular Component: plasmodesma (GO:0009506); Biological Process: response to salt stress (GO:0009651); Biological Process: embryo development ending in seed dormancy (GO:0009793); Biological Process: cell growth (GO:0016049); Biological Process: cysteine biosynthetic process (GO:0019344); Biological Process: response to cadmium ion (GO:0046686); Biological Process: Golgi vesicle transport (GO:0048193); | -- |
| BnaC08g41720D | 1.78E-15 | -∞ | Molecular Function: aspartic-type endopeptidase activity (GO:0004190); Cellular Component: extracellular region (GO:0005576); Cellular Component: vacuole (GO:0005773); Cellular Component: cytosol (GO:0005829); Biological Process: glycolytic process (GO:0006096); Biological Process: proteolysis (GO:0006508); Biological Process: protein targeting to vacuole (GO:0006623); Biological Process: lipid metabolic process (GO:0006629); Biological Process: water transport (GO:0006833); Biological Process: hyperosmotic response (GO:0006972); Biological Process: Golgi organization (GO:0007030); Biological Process: response to temperature stimulus (GO:0009266); Cellular Component: plasmodesma (GO:0009506); Biological Process: response to salt stress (GO:0009651); Biological Process: response to cadmium ion (GO:0046686); Biological Process: organ development (GO:0048513); | -- |
| BnaC08g41540D | 5.04E-15 | -∞ | Molecular Function: N,N-dimethylaniline monooxygenase activity (GO:0004499); Cellular Component: nucleus (GO:0005634); Biological Process: glucosinolate biosynthetic process (GO:0019761); Molecular Function: flavin adenine dinucleotide binding (GO:0050660); Molecular Function: NADP binding (GO:0050661); Biological Process: oxidation-reduction process (GO:0055114); Molecular Function: 8-methylthiopropyl glucosinolate S-oxygenase activity (GO:0080107); | -- |
| Cole_newGene_2243 | 1.33E-13 | -∞ | Cellular Component: mitochondrion (GO:0005739); Cellular Component: Golgi apparatus (GO:0005794); Cellular Component: plasma membrane (GO:0005886); Biological Process: response to stress (GO:0006950); Cellular Component: integral component of endoplasmic reticulum membrane (GO:0030176); Biological Process: single-organism cellular process (GO:0044763); | Protein export (ko03060); Protein processing in endoplasmic reticulum (ko04141) |
| Cole_newGene_1990 | 6.92E-08 | -∞ | -- | -- |
| BnaC04g10370D | 2.57E-17 | -∞ | Molecular Function: protein kinase activity (GO:0004672); Biological Process: phosphorylation (GO:0016310); | -- |
| Cole_newGene_1984 | 1.73E-22 | -∞ | Cellular Component: plant-type vacuole (GO:0000325); Cellular Component: vacuolar membrane (GO:0005774); Cellular Component: chloroplast (GO:0009507); Biological Process: response to salt stress (GO:0009651); Biological Process: proton transport (GO:0015992); Biological Process: cellular component organization (GO:0016043); Molecular Function: pyrophosphatase activity (GO:0016462); Molecular Function: transmembrane transporter activity (GO:0022857); Biological Process: ion transmembrane transport (GO:0034220); Biological Process: ATP metabolic process (GO:0046034); Biological Process: maintenance of location (GO:0051235); Biological Process: cellular localization (GO:0051641); Biological Process: cellular response to stimulus (GO:0051716); | Oxidative phosphorylation (ko00190); Phagosome (ko04145) |
| BnaC08g36200D | 3.79E-129 | -∞ | Cellular Component: chloroplast (GO:0009507); Biological Process: photorespiration (GO:0009853); | -- |
| BnaC08g29060D | 1.29E-11 | -∞ | Biological Process: RNA splicing, via endonucleolytic cleavage and ligation (GO:0000394); Molecular Function: inositol hexakisphosphate binding (GO:0000822); Biological Process: response to molecule of bacterial origin (GO:0002237); Molecular Function: ubiquitin-protein transferase activity (GO:0004842); Cellular Component: nucleus (GO:0005634); Cellular Component: vacuolar membrane (GO:0005774); Biological Process: methionine biosynthetic process (GO:0009086); Biological Process: auxin-activated signaling pathway (GO:0009734); Molecular Function: auxin binding (GO:0010011); Biological Process: stomatal complex morphogenesis (GO:0010103); Biological Process: pollen maturation (GO:0010152); Biological Process: protein ubiquitination (GO:0016567); Biological Process: stamen development (GO:0048443); Biological Process: lateral root development (GO:0048527); Biological Process: photoperiodism, flowering (GO:0048573); Biological Process: cellular response to nitrate (GO:0071249); Biological Process: primary root development (GO:0080022); | -- |
| BnaC05g27130D | 1.33E-11 | -∞ | -- | -- |
| BnaC05g26860D | 1.70E-11 | -∞ | -- | DNA replication (ko03030); Nucleotide excision repair (ko03420); Mismatch repair (ko03430); Homologous recombination (ko03440) |
| BnaA09g45320D | 4.72E-105 | -∞ | Molecular Function: copper ion binding (GO:0005507); Molecular Function: calmodulin binding (GO:0005516); Molecular Function: ATP binding (GO:0005524); Cellular Component: mitochondrion (GO:0005739); Cellular Component: cytosol (GO:0005829); Biological Process: gluconeogenesis (GO:0006094); Biological Process: glycolytic process (GO:0006096); Biological Process: protein folding (GO:0006457); Biological Process: tryptophan catabolic process (GO:0006569); Biological Process: response to heat (GO:0009408); Biological Process: response to cold (GO:0009409); Cellular Component: chloroplast thylakoid membrane (GO:0009535); Cellular Component: chloroplast stroma (GO:0009570); Biological Process: response to high light intensity (GO:0009644); Biological Process: response to salt stress (GO:0009651); Biological Process: chloroplast organization (GO:0009658); Biological Process: indoleacetic acid biosynthetic process (GO:0009684); Cellular Component: chloroplast envelope (GO:0009941); Biological Process: isopentenyl diphosphate biosynthetic process, methylerythritol 4-phosphate pathway (GO:0019288); Biological Process: cysteine biosynthetic process (GO:0019344); Biological Process: response to endoplasmic reticulum stress (GO:0034976); Biological Process: response to hydrogen peroxide (GO:0042542); Biological Process: response to cadmium ion (GO:0046686); Cellular Component: apoplast (GO:0048046); Biological Process: ovule development (GO:0048481); Molecular Function: chaperone binding (GO:0051087); Biological Process: positive regulation of superoxide dismutase activity (GO:1901671); | -- |
| BnaC08g40740D | 2.13E-05 | -∞ | Molecular Function: translation initiation factor activity (GO:0003743); Cellular Component: cytoplasm (GO:0005737); | -- |
| BnaA05g14900D | 0.000267257 | -∞ | Molecular Function: protein binding (GO:0005515); Cellular Component: nucleus (GO:0005634); Cellular Component: cytosol (GO:0005829); | -- |
