# Supplementary material for: Investigation of Thermomorphogenesis-Related Genes for a Multi-Silique Trait in Brassica napus by Comparative Transcriptome Analysis
Source: Front Genet. 2021 Jul 23;12:678804. doi: 10.3389/fgene.2021.678804 (PMC8343136; doi:10.3389/fgene.2021.678804)
Supplement: Supplementary Table 3 — Line-specific expressed genes from Ma’erkang. [file Table_3.DOCX]

**Supplementary Table 3|** Line-specific expressed genes from Ma’erkang.

| Gene ID | FDR | log_2_FC | GO annotation | KEGG pathway annotation |
| --- | --- | --- | --- | --- |
| BnaA10g10690D | 7.40E-10 | +∞ | Molecular Function: ATP transmembrane transporter activity (GO:0005347); Cellular Component: mitochondrial inner membrane (GO:0005743); Biological Process: mitochondrial transport (GO:0006839); Biological Process: anther development (GO:0048653); | -- |
| BnaAnng17190D | 2.15E-18 | +∞ | Biological Process: maltose metabolic process (GO:0000023); Biological Process: sucrose metabolic process (GO:0005985); Biological Process: fructose metabolic process (GO:0006000); Biological Process: pentose-phosphate shunt (GO:0006098); Biological Process: rRNA processing (GO:0006364); Biological Process: response to cold (GO:0009409); Cellular Component: chloroplast stroma (GO:0009570); Biological Process: response to blue light (GO:0009637); Biological Process: photosynthetic electron transport in photosystem I (GO:0009773); Biological Process: chloroplast relocation (GO:0009902); Biological Process: thylakoid membrane organization (GO:0010027); Biological Process: response to red light (GO:0010114); Biological Process: photosystem II assembly (GO:0010207); Biological Process: response to far red light (GO:0010218); Cellular Component: stromule (GO:0010319); Biological Process: chlorophyll biosynthetic process (GO:0015995); Biological Process: carotenoid biosynthetic process (GO:0016117); Biological Process: starch biosynthetic process (GO:0019252); Biological Process: reductive pentose-phosphate cycle (GO:0019253); Biological Process: cellular cation homeostasis (GO:0030003); Biological Process: fructose 1,6-bisphosphate metabolic process (GO:0030388); Biological Process: regulation of protein dephosphorylation (GO:0035304); Molecular Function: fructose 1,6-bisphosphate 1-phosphatase activity (GO:0042132); Biological Process: defense response to bacterium (GO:0042742); Biological Process: positive regulation of catalytic activity (GO:0043085); Molecular Function: metal ion binding (GO:0046872); Cellular Component: apoplast (GO:0048046); Biological Process: divalent metal ion transport (GO:0070838); | Glycolysis / Gluconeogenesis (ko00010); Pentose phosphate pathway (ko00030); Fructose and mannose metabolism (ko00051); Carbon fixation in photosynthetic organisms (ko00710); Carbon metabolism (ko01200) |
| BnaAnng19640D | 5.03E-13 | +∞ | -- | -- |
| BnaC02g05120D | 1.10E-19 | +∞ | Cellular Component: cell wall (GO:0005618); Cellular Component: mitochondrial inner membrane (GO:0005743); Cellular Component: vacuolar membrane (GO:0005774); Cellular Component: Golgi apparatus (GO:0005794); Biological Process: pentose-phosphate shunt (GO:0006098); Biological Process: transport (GO:0006810); Cellular Component: chloroplast (GO:0009507); Biological Process: response to salt stress (GO:0009651); Cellular Component: integral component of membrane (GO:0016021); | -- |
| BnaC04g06070D | 1.75E-08 | +∞ | -- | -- |
| BnaC08g35720D | 7.53E-07 | +∞ | Cellular Component: vacuolar proton-transporting V-type ATPase, V0 domain (GO:0000220); Cellular Component: mitochondrion (GO:0005739); Cellular Component: Golgi apparatus (GO:0005794); Biological Process: obsolete ATP catabolic process (GO:0006200); Cellular Component: chloroplast (GO:0009507); Molecular Function: hydrogen-translocating pyrophosphatase activity (GO:0009678); Cellular Component: plant-type vacuole membrane (GO:0009705); Molecular Function: hydrogen ion transmembrane transporter activity (GO:0015078); Biological Process: ATP synthesis coupled proton transport (GO:0015986); Biological Process: ATP hydrolysis coupled proton transport (GO:0015991); Molecular Function: ATPase activity (GO:0016887); Biological Process: cellular response to nutrient levels (GO:0031669); Biological Process: sequestering of zinc ion (GO:0032119); Biological Process: vacuolar sequestering (GO:0043181); Molecular Function: nutrient reservoir activity (GO:0045735); Biological Process: vacuolar proton-transporting V-type ATPase complex assembly (GO:0070072); Biological Process: cellular response to salt stress (GO:0071472); | Oxidative phosphorylation (ko00190); Phagosome (ko04145) |
| BnaC08g36570D | 1.44E-36 | +∞ | Molecular Function: actin binding (GO:0003779); Cellular Component: cell wall (GO:0005618); Cellular Component: nucleolus (GO:0005730); Cellular Component: spindle (GO:0005819); Cellular Component: cytosol (GO:0005829); Cellular Component: plasma membrane (GO:0005886); Biological Process: actin polymerization or depolymerization (GO:0008154); Cellular Component: plasmodesma (GO:0009506); Cellular Component: chloroplast (GO:0009507); Cellular Component: phragmoplast (GO:0009524); Biological Process: unidimensional cell growth (GO:0009826); Cellular Component: actin cytoskeleton (GO:0015629); Cellular Component: apoplast (GO:0048046); | -- |
| BnaC08g39040D | 2.97E-12 | +∞ | Molecular Function: CDP-diacylglycerol-serine O-phosphatidyltransferase activity (GO:0003882); Cellular Component: nucleus (GO:0005634); Cellular Component: mitochondrion (GO:0005739); Cellular Component: endoplasmic reticulum membrane (GO:0005789); Biological Process: phosphatidylserine biosynthetic process (GO:0006659); | Glycerophospholipid metabolism (ko00564) |
| BnaC08g39120D | 8.67E-11 | +∞ | -- | -- |
| BnaC08g39130D | 2.37E-29 | +∞ | Molecular Function: copper ion binding (GO:0005507); Molecular Function: calmodulin binding (GO:0005516); Molecular Function: ATP binding (GO:0005524); Cellular Component: mitochondrion (GO:0005739); Cellular Component: cytosol (GO:0005829); Biological Process: gluconeogenesis (GO:0006094); Biological Process: glycolytic process (GO:0006096); Biological Process: protein folding (GO:0006457); Biological Process: tryptophan catabolic process (GO:0006569); Biological Process: response to heat (GO:0009408); Biological Process: response to cold (GO:0009409); Cellular Component: chloroplast thylakoid membrane (GO:0009535); Cellular Component: chloroplast stroma (GO:0009570); Biological Process: response to high light intensity (GO:0009644); Biological Process: response to salt stress (GO:0009651); Biological Process: chloroplast organization (GO:0009658); Biological Process: indoleacetic acid biosynthetic process (GO:0009684); Cellular Component: chloroplast envelope (GO:0009941); Biological Process: isopentenyl diphosphate biosynthetic process, methylerythritol 4-phosphate pathway (GO:0019288); Biological Process: cysteine biosynthetic process (GO:0019344); Biological Process: response to endoplasmic reticulum stress (GO:0034976); Biological Process: response to hydrogen peroxide (GO:0042542); Biological Process: response to cadmium ion (GO:0046686); Cellular Component: apoplast (GO:0048046); Biological Process: plant ovule development (GO:0048481); Molecular Function: chaperone binding (GO:0051087); Biological Process: positive regulation of superoxide dismutase activity (GO:1901671); | -- |
| BnaC08g39360D | 1.14E-06 | +∞ | Molecular Function: hydrolase activity, hydrolyzing O-glycosyl compounds (GO:0004553); Cellular Component: cell wall (GO:0005618); Biological Process: cellular glucan metabolic process (GO:0006073); Biological Process: phloem or xylem histogenesis (GO:0010087); Biological Process: fruit development (GO:0010154); Molecular Function: xyloglucan:xyloglucosyl transferase activity (GO:0016762); Cellular Component: apoplast (GO:0048046); Biological Process: stamen filament development (GO:0080086); | -- |
| BnaC08g40410D | 2.05E-22 | +∞ | Molecular Function: GTPase activator activity (GO:0005098); Cellular Component: nuclear envelope (GO:0005635); Cellular Component: vacuolar membrane (GO:0005774); Cellular Component: endoplasmic reticulum (GO:0005783); Biological Process: nucleocytoplasmic transport (GO:0006913); Biological Process: toxin catabolic process (GO:0009407); Cellular Component: chloroplast (GO:0009507); Biological Process: photomorphogenesis (GO:0009640); Biological Process: response to salt stress (GO:0009651); Biological Process: protein deneddylation (GO:0010388); Biological Process: lateral root development (GO:0048527); | RNA transport (ko03013) |
| BnaC09g05400D | 2.10E-14 | +∞ | Cellular Component: nucleus (GO:0005634); | -- |
| BnaC09g06260D | 1.07E-31 | +∞ | Cellular Component: nucleus (GO:0005634); | -- |
| BnaC09g53990D | 3.02E-23 | +∞ | Biological Process: sulfur amino acid metabolic process (GO:0000096); Biological Process: MAPK cascade (GO:0000165); Molecular Function: iron ion binding (GO:0005506); Cellular Component: nucleus (GO:0005634); Biological Process: pentose-phosphate shunt (GO:0006098); Biological Process: regulation of translation (GO:0006417); Biological Process: glycine catabolic process (GO:0006546); Biological Process: protein targeting to membrane (GO:0006612); Biological Process: unsaturated fatty acid biosynthetic process (GO:0006636); Biological Process: phosphatidylglycerol biosynthetic process (GO:0006655); Biological Process: vitamin metabolic process (GO:0006766); Biological Process: cellular amino acid biosynthetic process (GO:0008652); Biological Process: aromatic amino acid family metabolic process (GO:0009072); Biological Process: lipoate metabolic process (GO:0009106); Biological Process: coenzyme biosynthetic process (GO:0009108); Biological Process: response to cold (GO:0009409); Cellular Component: chloroplast stroma (GO:0009570); Biological Process: detection of biotic stimulus (GO:0009595); Biological Process: response to blue light (GO:0009637); Biological Process: response to high light intensity (GO:0009644); Biological Process: plastid organization (GO:0009657); Biological Process: jasmonic acid biosynthetic process (GO:0009695); Biological Process: salicylic acid biosynthetic process (GO:0009697); Biological Process: response to sucrose (GO:0009744); Biological Process: systemic acquired resistance, salicylic acid mediated signaling pathway (GO:0009862); Biological Process: jasmonic acid mediated signaling pathway (GO:0009867); Cellular Component: chloroplast envelope (GO:0009941); Biological Process: response to red light (GO:0010114); Biological Process: regulation of proton transport (GO:0010155); Biological Process: response to chitin (GO:0010200); Biological Process: response to far red light (GO:0010218); Biological Process: PSII associated light-harvesting complex II catabolic process (GO:0010304); Biological Process: regulation of hydrogen peroxide metabolic process (GO:0010310); Biological Process: regulation of plant-type hypersensitive response (GO:0010363); Biological Process: chlorophyll biosynthetic process (GO:0015995); Biological Process: carotenoid biosynthetic process (GO:0016117); Biological Process: regulation of lipid metabolic process (GO:0019216); Biological Process: starch biosynthetic process (GO:0019252); Biological Process: isopentenyl diphosphate biosynthetic process, methylerythritol 4-phosphate pathway (GO:0019288); Biological Process: photosynthesis, light reaction (GO:0019684); Biological Process: glucosinolate metabolic process (GO:0019760); Biological Process: negative regulation of defense response (GO:0031348); Biological Process: oxylipin biosynthetic process (GO:0031408); Biological Process: defense response to bacterium (GO:0042742); Biological Process: regulation of multi-organism process (GO:0043900); Biological Process: sulfur compound biosynthetic process (GO:0044272); Biological Process: positive regulation of transcription, DNA-templated (GO:0045893); Molecular Function: 4-hydroxy-3-methylbut-2-en-1-yl diphosphate synthase activity (GO:0046429); Biological Process: defense response to fungus (GO:0050832); Molecular Function: 4 iron, 4 sulfur cluster binding (GO:0051539); | Terpenoid backbone biosynthesis (ko00900) |
| Brassica_napus_newGene_12095 | 4.96E-17 | +∞ | Cellular Component: cell wall (GO:0005618); Cellular Component: vacuole (GO:0005773); Cellular Component: endoplasmic reticulum (GO:0005783); Cellular Component: plasma membrane (GO:0005886); Cellular Component: plasmodesma (GO:0009506); Cellular Component: integral component of membrane (GO:0016021); Molecular Function: transmembrane transporter activity (GO:0022857); Biological Process: transmembrane transport (GO:0055085); | -- |
| Brassica_napus_newGene_3308 | 3.34E-23 | +∞ | Molecular Function: ATP binding (GO:0005524); Cellular Component: cell wall (GO:0005618); Cellular Component: mitochondrion (GO:0005739); Cellular Component: chloroplast stroma (GO:0009570); | Protein processing in endoplasmic reticulum (ko04141); Plant-pathogen interaction (ko04626) |
| Brassica_napus_newGene_8007 | 1.24E-13 | +∞ | -- | -- |
| BnaA01g22780D | 1.07E-20 | -∞ | Molecular Function: transcription coactivator activity (GO:0003713); Cellular Component: cytoplasm (GO:0005737); Molecular Function: zinc ion binding (GO:0008270); | -- |
