# Supplementary material for: Investigation of Thermomorphogenesis-Related Genes for a Multi-Silique Trait in Brassica napus by Comparative Transcriptome Analysis
Source: Front Genet. 2021 Jul 23;12:678804. doi: 10.3389/fgene.2021.678804 (PMC8343136; doi:10.3389/fgene.2021.678804)
Supplement: Supplementary Table 4 — The 33 stable DEGs with the same or similar expression tendency in two environments. [file Table_4.DOCX]

**Supplementary Table 4|** The 33 stable DEGs with the same or similar expression tendency in two environments.

| Gene ID | log_2_FC in Xindu | log_2_FC in Ma’erkang | regulated |
| --- | --- | --- | --- |
| BnaA01g10540D | -4.532974033 | -2.32916 | down |
| BnaA03g35870D | 4.148572674 | 2.542677 | up |
| BnaA07g04500D | 2.610401631 | 3.568468 | up |
| BnaA09g06740D | +∞ | 7.570671 | up |
| BnaA09g48320D | -9.731591451 | -2.64282 | down |
| BnaAnng30260D | 4.006054633 | 3.71287 | up |
| BnaC01g02500D | 5.61655146 | 5.877495 | up |
| BnaC01g43270D | +∞ | 4.066506 | up |
| BnaC02g06410D | -∞ | -2.98529 | down |
| BnaC03g57080D | 3.813480654 | 3.602956 | up |
| BnaC03g65980D | -5.041401489 | -4.78954 | down |
| BnaC06g16950D | 3.198110463 | 3.968274 | up |
| BnaC07g36960D | 2.925531986 | 4.294565 | up |
| BnaC08g01470D | 4.755368345 | 4.522446 | up |
| BnaC08g35720D | +∞ | +∞ | up |
| BnaC08g36200D | -∞ | -2.68255 | down |
| BnaC08g38300D | -∞ | -3.195 | down |
| BnaC08g39120D | +∞ | +∞ | up |
| BnaC08g39130D | +∞ | +∞ | up |
| BnaC08g39990D | 2.712907065 | 4.097042 | up |
| BnaC08g40410D | +∞ | +∞ | up |
| BnaC08g40810D | 3.048041065 | 2.406417 | up |
| BnaC08g41180D | -2.721461203 | -2.5698 | down |
| BnaC08g41720D | -∞ | -2.69705 | down |
| BnaC08g42280D | -∞ | -3.798 | down |
| BnaC08g49610D | -8.084367201 | -2.84759 | down |
| BnaC09g05590D | +∞ | 2.881326 | up |
| BnaC09g05960D | +∞ | 5.221473 | up |
| BnaC09g06220D | 7.040607444 | 4.951949 | up |
| BnaC09g06260D | 6.944273498 | +∞ | up |
| BnaCnng17490D | 3.703814625 | 3.632534 | up |
| BnaCnng24040D | 4.770311741 | 4.922701 | up |
| BnaCnng36050D | 2.457334101 | 3.7552 | up |
