# Supplementary material for: Investigation of Thermomorphogenesis-Related Genes for a Multi-Silique Trait in Brassica napus by Comparative Transcriptome Analysis
Source: Front Genet. 2021 Jul 23;12:678804. doi: 10.3389/fgene.2021.678804 (PMC8343136; doi:10.3389/fgene.2021.678804)
Supplement: Supplementary Table 5 — The 96 environment-specific DEGs with different expression tendency in two environments. [file Table_5.DOCX]

**Supplementary Table 5|** The 96 environment-specific DEGs with different expression tendency in two environments.

| Gene ID | Xindu | |  | Ma'erkang | |
| --- | --- | --- | --- | --- | --- |
|  | log_2_FC | regulated |  | log_2_FC | regulated |
| BnaA01g26720D | -8.803041102 | down |  | -- | -- |
| BnaA02g02630D | -2.017932931 | down |  | -0.31717265 | normal |
| BnaA02g03080D | -2.073352665 | down |  | -1.651613138 | normal |
| BnaA04g00770D | -2.196007248 | down |  | -1.367605254 | normal |
| BnaA04g06410D | +∞ | up |  | -- | -- |
| BnaA04g16220D | -2.102568537 | down |  | -0.768495114 | normal |
| BnaA05g14900D | -∞ | down |  | -0.294007816 | normal |
| BnaA05g21710D | 3.115269184 | up |  | 0.926207134 | normal |
| BnaA07g00820D | 2.528682859 | up |  | -- | -- |
| BnaA07g09660D | 6.394250737 | up |  | -- | -- |
| BnaA07g27870D | 4.710001097 | up |  | -- | -- |
| BnaA08g02930D | 2.176144806 | up |  | -- | -- |
| BnaA09g43250D | 4.607141719 | up |  | 1.792865026 | normal |
| BnaA09g44370D | -2.361493162 | down |  | -1.122441591 | normal |
| BnaA09g44650D | -3.105926356 | down |  | -- | -- |
| BnaA09g45000D | -2.666365322 | down |  | -1.250098756 | normal |
| BnaA09g45260D | -2.144652279 | down |  | -0.591314237 | normal |
| BnaA09g45300D | -5.750255404 | down |  | -- | -- |
| BnaA09g45310D | 2.352510084 | up |  | -- | -- |
| BnaA09g45320D | -∞ | down |  | -2.401372121 | normal |
| BnaA09g45610D | 3.440667186 | up |  | -- | -- |
| BnaA09g45890D | -3.091510192 | down |  | -- | -- |
| BnaA09g46080D | -4.45666296 | down |  | -- | -- |
| BnaA09g47900D | -∞ | down |  | -- | -- |
| BnaA09g56410D | -2.049715623 | down |  | -0.967409204 | normal |
| BnaA09g56740D | -2.223147044 | down |  | -- | -- |
| BnaA10g00400D | 2.205407167 | up |  | 0.828051411 | normal |
| BnaA10g07970D | -2.910933074 | down |  | -- | -- |
| BnaAnng13790D | 5.034996768 | up |  | 2.454890235 | normal |
| BnaAnng14640D | 2.37283138 | up |  | -- | -- |
| BnaAnng35580D | -2.652367352 | down |  | -0.430432427 | normal |
| BnaC02g06360D | -∞ | down |  | -- | -- |
| BnaC02g06440D | -2.042831435 | down |  | -- | -- |
| BnaC02g06570D | -∞ | down |  | -2.952049933 | normal |
| BnaC03g09190D | -4.131346977 | down |  | -- | -- |
| BnaC03g19830D | 3.42720008 | up |  | -- | -- |
| BnaC03g24650D | -∞ | down |  | -- | -- |
| BnaC03g63980D | 3.373910018 | up |  | 0.856273786 | normal |
| BnaC03g76890D | 3.898103597 | up |  | 0.231902422 | normal |
| BnaC04g10370D | -∞ | down |  | -2.490506247 | normal |
| BnaC04g29730D | +∞ | up |  | -- | -- |
| BnaC04g30180D | +∞ | up |  | -- | -- |
| BnaC04g30490D | +∞ | up |  | -- | -- |
| BnaC04g39120D | -2.750227203 | down |  | -0.949307593 | normal |
| BnaC04g45730D | 2.322679988 | up |  | -- | -- |
| BnaC05g26860D | -∞ | down |  | -- | -- |
| BnaC05g27130D | -∞ | down |  | -- | -- |
| BnaC05g49350D | -2.579757131 | down |  | 0.181823956 | normal |
| BnaC06g07110D | 4.02437143 | up |  | -- | -- |
| BnaC06g10430D | -4.041118501 | down |  | -- | -- |
| BnaC06g42000D | 3.532449954 | up |  | 2.693834066 | normal |
| BnaC07g33980D | 2.904839217 | up |  | 1.393879506 | normal |
| BnaC08g29060D | -∞ | down |  | -2.22474113 | normal |
| BnaC08g35850D | 4.962240429 | up |  | -- | -- |
| BnaC08g35880D | -2.360925007 | down |  | -0.641705277 | normal |
| BnaC08g36100D | -3.04970056 | down |  | -- | -- |
| BnaC08g36360D | 3.158331419 | up |  | 3.677830226 | normal |
| BnaC08g37340D | +∞ | up |  | -- | -- |
| BnaC08g37460D | -7.289243271 | down |  | -2.439435452 | normal |
| BnaC08g38200D | -2.794757281 | down |  | -- | -- |
| BnaC08g39020D | +∞ | up |  | -- | -- |
| BnaC08g40320D | +∞ | up |  | -- | -- |
| BnaC08g40740D | -∞ | down |  | -- | -- |
| BnaC08g41390D | -∞ | down |  | -1.545012913 | normal |
| BnaC08g41540D | -∞ | down |  | -2.213542307 | normal |
| BnaC08g41780D | -∞ | down |  | -- | -- |
| BnaC08g42080D | -4.450250437 | down |  | -1.615979135 | normal |
| BnaC08g42450D | -4.027913366 | down |  | -- | -- |
| BnaC08g49500D | +∞ | up |  | -- | -- |
| BnaC09g06110D | +∞ | up |  | -- | -- |
| BnaCnng49710D | -3.755203226 | down |  | -- | -- |
| BnaCnng68410D | 4.58740867 | up |  | -- | -- |
| BnaCnng75420D | +∞ | up |  | -- | -- |
| Cole_newGene_1596 | -8.232679558 | down |  | -- | -- |
| Cole_newGene_1717 | -5.638144992 | down |  | -- | -- |
| Cole_newGene_1891 | 2.711544687 | up |  | -- | -- |
| Cole_newGene_1939 | 3.735730389 | up |  | -- | -- |
| Cole_newGene_1983 | -∞ | down |  | -- | -- |
| Cole_newGene_1984 | -∞ | down |  | -- | -- |
| Cole_newGene_1990 | -∞ | down |  | -- | -- |
| Cole_newGene_2071 | -∞ | down |  | -- | -- |
| Cole_newGene_2073 | +∞ | up |  | -- | -- |
| Cole_newGene_2243 | -∞ | down |  | -- | -- |
| Cole_newGene_2682 | +∞ | up |  | -- | -- |
| Cole_newGene_269 | 3.770106258 | up |  | -- | -- |
| Cole_newGene_2756 | 2.932403275 | up |  | -- | -- |
| Cole_newGene_3294 | 2.842935963 | up |  | -- | -- |
| Cole_newGene_3682 | -2.272637253 | down |  | -- | -- |
| Cole_newGene_3766 | -8.356368747 | down |  | -- | -- |
| Cole_newGene_4151 | -∞ | down |  | -- | -- |
| Cole_newGene_4761 | 2.480454995 | up |  | -- | -- |
| Cole_newGene_5614 | 5.864268051 | up |  | -- | -- |
| Cole_newGene_6035 | 5.929928585 | up |  | -- | -- |
| Cole_newGene_6107 | 4.144976781 | up |  | -- | -- |
| Cole_newGene_6687 | +∞ | up |  | -- | -- |
| Cole_newGene_959 | 3.359629129 | up |  | -- | -- |
